# *Thermotoga maritima oriC* involves a DNA unwinding element with distinct modules and a DnaA-oligomerizing region with a novel directional binding mode

**DOI:** 10.1016/j.jbc.2023.104888

**Published:** 2023-06-03

**Authors:** Chuyuan Lu, Ryusei Yoshida, Tsutomu Katayama, Shogo Ozaki

**Affiliations:** Department of Molecular Biology, Graduate School of Pharmaceutical Sciences, Kyushu University, Higashi-ku, Fukuoka, Japan

**Keywords:** AAA+, DnaA, DNA replication, DNA–protein interaction, DNA unwinding, *oriC*, protein–protein interaction

## Abstract

Initiation of chromosomal replication requires dynamic nucleoprotein complexes. In most eubacteria, the origin *oriC* contains multiple DnaA box sequences to which the ubiquitous DnaA initiators bind. In *Escherichia coli oriC*, DnaA boxes sustain construction of higher-order complexes *via* DnaA–DnaA interactions, promoting the unwinding of the DNA unwinding element (DUE) within *oriC* and concomitantly binding the single-stranded (ss) DUE to install replication machinery. Despite the significant sequence homologies among DnaA proteins, *oriC* sequences are highly diverse. The present study investigated the design of *oriC* (*tma-oriC*) from *Thermotoga maritima*, an evolutionarily ancient eubacterium. The minimal *tma-oriC* sequence includes a DUE and a flanking region containing five DnaA boxes recognized by the cognate DnaA (*tma*DnaA). This DUE was comprised of two distinct functional modules, an unwinding module and a *tma*DnaA-binding module. Three direct repeats of the trinucleotide TAG within DUE were essential for both unwinding and ssDUE binding by *tma*DnaA complexes constructed on the DnaA boxes. Its surrounding AT-rich sequences stimulated only duplex unwinding. Moreover, head-to-tail oligomers of ATP-bound *tma*DnaA were constructed within *tma-oriC*, irrespective of the directions of the DnaA boxes. This binding mode was considered to be induced by flexible swiveling of DnaA domains III and IV, which were responsible for DnaA–DnaA interactions and DnaA box binding, respectively. Phasing of specific *tma*DnaA boxes in *tma-oriC* was also responsible for unwinding. These findings indicate that a ssDUE recruitment mechanism was responsible for unwinding and would enhance understanding of the fundamental molecular nature of the origin sequences present in evolutionarily divergent bacteria.

Unwinding of duplex DNA is fundamental for chromosomal DNA replication in all cellular organisms. The origin of replication *oriC* encodes instructions to form a highly ordered nucleoprotein complex called the initiation complex. In bacteria, this complex is mainly comprised of the ubiquitous family of DnaA initiator proteins (1, 2, 3, 4, 5, 6). The minimal *oriC* in *Escherichia coli* consists of an AT-rich DNA unwinding element (DUE) and a flanking DnaA oligomerization region (DOR) containing a cluster of DnaA-binding sequences (DnaA boxes) (Fig. 1*A*). The ATP-bound DnaA oligomers of the DOR are important for DUE unwinding and loading of DnaB replicative helicase onto the unwound region (5, 7, 8, 9, 10, 11).

**Figure 1:**
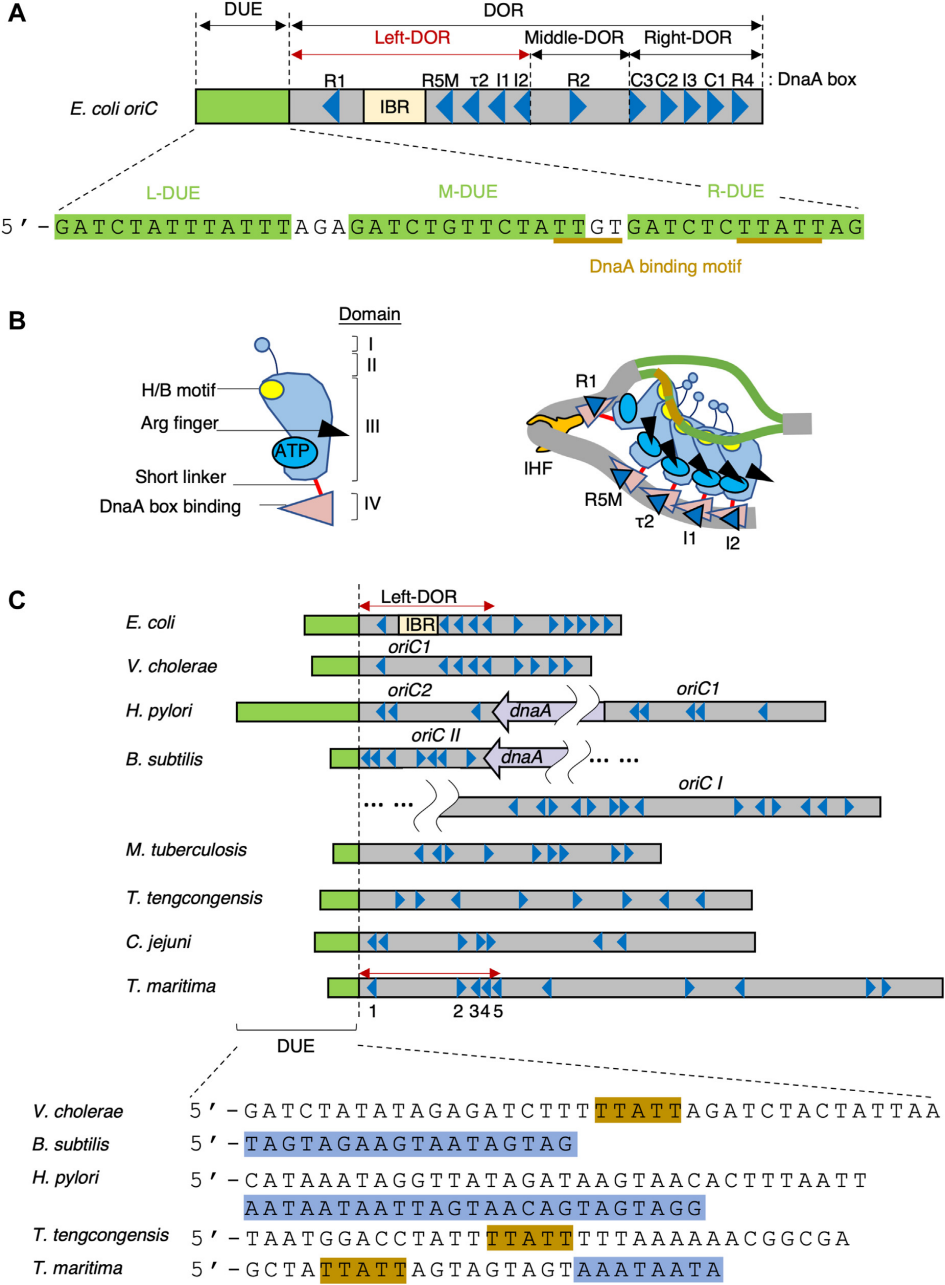
**Models for unwinding of the replication origin in eubacteria**. *A*, the structure of *E. coli oriC*. DnaA boxes (R1, R5M, τ2, I1, I2, C3, C2, I3, C1, and R4) are indicated by *arrowheads* and the IHF-binding region (IBR) by a *rectangle*. DnaA box τ1, which overlaps IBR, is omitted for simplicity. DUE comprises L-, M-, and R-DUE (colored in *green*). The DnaA-binding motifs, TTGT and TTATT, are highlighted in *brown bars*. *Left* DOR spans DnaA boxes R1–I2, *middle* DOR consists of DnaA box R2, and *right* DOR spans DnaA boxes C3–R4. *B*, a model for open complex formation through the ssDUE recruitment mechanism. Schematic illustration of the domain architecture (I-IV) of a typical protein of the DnaA family. Domains III and IV are connected by a short linker in *E. coli*. The H/B and Arg-finger motifs are also indicated. In *E. coli*, the ATP-DnaA pentamer formed on IHF-bound left DOR unwinds DUE and concomitantly binds the upper strand of the ssDUE in a manner specific to the sequence TT[G/A]T(T). *C*, comparison of the overall structures of the replication origins of Eubacteria. DORs of the indicated eubacterial organisms are aligned. The bilobed structure of the origins of *Bacillus subtilis* and *Heliobacter pylori* are depicted, with one lobe, *oriC II* for *B. subtilis* and *oriC2* for *H. pylori*, bearing the DUE. The overall structures of the origins of *Mycobacterium tuberculosis* (29), *Thermoanaerobacter tengcongensis* (30), and *Camplyobacter jejuni* (31) are also shown. DUE is indicated by *green bars*, and the sequences of experimentally characterized DUEs from *B. subtilis H. pylori*, *T. tengcongensis*, and *Thermotoga maritima* are shown below, highlighting the TTATT motif in *brown* and DnaA-trios in *blue*. DnaA boxes and IBR are shown as in *panel A*. *E. coli* Left-DOR and minimal *tma*DOR are indicated by *red left-right arrows*. DOR, DnaA oligomerization region; DUE, duplex unwinding element; IHF, integration host factor; ss, single-stranded; *tma*, *Thermotoga maritima*.

Proteins in the DnaA family contain a central domain III with AAA+ (ATPases associated with various cellular activities) motifs (2, 4, 5, 12) (Fig. 1*B*). These motifs play essential roles in ATP/ADP binding, ATP hydrolysis, and DnaA–DnaA interactions (10, 12, 13). Similar to other proteins containing AAA+ motifs, head-to-tail oligomerization *via* this domain underlies the formation of an initiation complex by ATP–DnaA. In *E. coli* DnaA, the AAA+ arginine-finger motif Arg285 predominantly recognizes ATP bound to the adjacent DnaA protomer, promoting co-operative ATP-DnaA binding onto the DOR in a head-to-tail orientation (14, 15, 16) (Fig. 1*B*). Moreover, H/B-motifs (hydrophobic Val211 and basic Arg245) in this domain bind single-stranded DUEs (ssDUEs) in a sequence-specific manner (9, 17) (Fig. 1, *A* and *B*). The AAA+ domain III of DnaA is linked *via* a flexible hinge to its C-terminal domain IV, which is responsible for DnaA box-specific DNA binding (18)(Fig. 1*B*). The N-terminal domain I has multiple sites for interactions with DnaB helicase etc. and for weak domain I–domain I interactions (11, 14, 19, 20, 21, 22, 23). Domain II is a flexible linker between domains I and III (21, 24) (Fig. 1*B*).

The arrangement of DnaA boxes within the DOR provides an essential scaffold for formation of the initiation complex (Fig. 1, *A* and *C*). A canonical DnaA box consists of an asymmetric 9-mer consensus sequence, TTA[T/A]NCACA (3, 25). The DOR in *E. coli* contains no fewer than 12 DnaA boxes as well as a region for specific binding to integration host factor (IHF), a nucleoid-associated protein that introduces a sharp bend in DNA (IHF-binding region) (Fig. 1, *A* and *B*) (1, 2). Five of these DnaA boxes in the left DOR (R1, R5M, τ2, and I1-2) share the same orientation, whereas five boxes in the right DOR (R4, C1-3, and I3) share the opposite orientation (8, 14, 15, 26, 27, 28). The directional arrangement of the DnaA boxes facilitates head-to-tail oligomerization of ATP–DnaA proteins depending on the ATP–Arg finger interaction. This leads to formation of a pair of pentamers bound to the left and right DORs, with the two facing each other. In the left DOR, the IHF-dependent bending facilitates formation of the DnaA complexes, promoting DUE unwinding activity (26) (Fig. 1*B*). DnaA complexes in the right DOR are important for enhancement of the unwound state and efficient DnaB loading (7, 9).

Experimental determination of the *oriC*s of representative bacterial species has shown that the basic structure of *Vibrio cholerae oriC*1, the origin of chromosome I, is most similar to that of *E. coli oriC*, in that the DUE is flanked by a region containing two DnaA box clusters in the opposite directions (3) (Fig. 1*C*). The *oriC* of *Heliobacter pylori* is split into two subsequences, *oriC*1 and *oriC*2, by insertion of the *dnaA* gene; the *oriC*2 of *H. pylori* may correspond to the region of the *E. coli oriC* spanning DUE to the left DOR, a region containing a single cluster of unidirectional DnaA boxes (3) (Fig. 1*C*). However, the *oriC* sequences from other species vary in the directions of DnaA boxes, despite containing direct repeats of DnaA box sequences (29, 30, 31). Similar features have also been observed in the bioinformatically predicted *oriC*s of bacterial genomes (32) (Fig. S1). Thus, the principles underlying the designs of DnaA box arrangements within bacterial DORs that underlie the construction of DnaA oligomers remain unclear.

In *E. coli*, the ATP-DnaA–IHF–Left-DOR complexes are responsible for the unwinding of DUE. The upper strand of the resulting ssDUE is subsequently recruited to the ATP–DnaA pentamers through IHF binding-induced DOR bending, which directs interactions between the H/B-motifs of DnaA and ssDUE, resulting in open complex formation (7, 9, 17, 26) (Fig. 1, *A* and *B*). This ssDUE recruitment mechanism stabilizes the unwound state of DUE within the open complex, allowing efficient DnaB replicative helicase loading onto the ssDUE region. In-depth analyses have shown that the TTGT/TTATT motifs within DUE bind the DnaA–DOR complex and are crucial for the initiation of replication (17, 33) (Fig. 1, *B* and *C*), emphasizing the physiological importance of DnaA–ssDUE interactions.

Functional DnaA–ssDUE interactions have also been implicated in other bacterial species, including *Bacillus subtilis* and *H. pylori* (34, 35). Both origins display a bilobed structure, with *oriC*1 and *oriC*2 being separated by insertion of a *dnaA* gene. *B. subtilis oriC*2 carries the cognate DOR with seven DnaA boxes, followed by a GC-rich 5-mer and a flanking DUE region that includes 5′-TAG-TAG-AAG-TAA-TAG-TAG-3′ sequences (Fig. 1*C*). Of these sequences, the two adenine residues at positions 14 and 17 are crucial for *in vivo* initiation and repeats of the trinucleotide termed DnaA-trios, 5′-TAG-3′, 5′-TAA-3′, and 5′-AAG-3′, are contained (34, 36). Chemical cross-linking has indicated that the *B. subtilis* DnaA-trios on ssDUE promote the oligomerization of cognate ATP-DnaA, depending on the DnaA bound to the duplex DNA DnaA boxes flanking the DUE, thereby supporting DUE unwinding *in vitro* (34, 36). By contrast, in *H. pylori oriC2* carrying the DUE with the cognate DnaA-trios (5′-AAT-AAT-AAT-TAG-TAA-CAG-TAG-TAG-3′), the cognate DnaA–DOR complexes bind the ssDUE (35) (Fig. 1*C*). Similar mechanisms involving ssDUE interactions of the initiator protein have been observed for the *V. cholerae* chromosome 2 origin (*oriC2*) and its cognate initiator RctB, although RctB is not an AAA+ protein (37). *oriC2* carries the cognate DUE and the flanking regions with multiple RctB-binding sequences. RctB oligomers within *oriC2* interact with ATCA repeats of ssDUE in a manner stimulated by IHF binding and DNA looping between the two RctB-interacting regions. These mechanisms in *H. pylori* and *V. cholerae* were principally similar to the ssDUE recruitment mechanism in *E. coli* (Fig. 1*B*).

The ssDUE recruitment mechanism has also been observed at the origin of replication of the bacterium *Thermotoga maritima* (*tma-oriC*). Phylogenetic and biological analyses have placed this organism at a deep branch in the tree of life (38, 39), with its DnaA initiator (*tma*DnaA) recognizing the noncanonical DnaA box with an asymmetric sequence (*tma*DnaA box) repeated 10 times within *tma-oriC* (Figs. 1*C* and S2). The 149 bp minimal *tma-oriC*, which is responsible for specific unwinding, contains a 24 bp AT-rich *tma*DUE and a flanking *tma*DOR consisting of *tma*DnaA boxes 1 to 5 (40) (Fig. 2*A*). Although *tma*DnaA box 2 is oriented in the opposite direction, the *tma*DOR is associated with the formation of ATP-*tma*DnaA oligomers responsible for DUE unwinding. Moreover, the upper strand of *tma*-ssDUE binds to ATP-*tma*DnaA oligomers bound to *tma*DOR, depending on the *tma*DnaA residues Val176 and Lys209, which correspond to the H/B-motifs (17). These observations are in good agreement with the ssDUE recruitment mechanism. Notably, *tma*DnaA boxes 3 to 5 play a crucial role in formation of ATP-*tma*DnaA oligomers responsible for ssDUE binding (9). However, the contribution of the oppositely oriented *tma*DnaA box 2 to the formation of the initiation complex remains unclear, as do the *tma*DUE sequences responsible for DUE unwinding and ssDUE binding.

**Figure 2:**
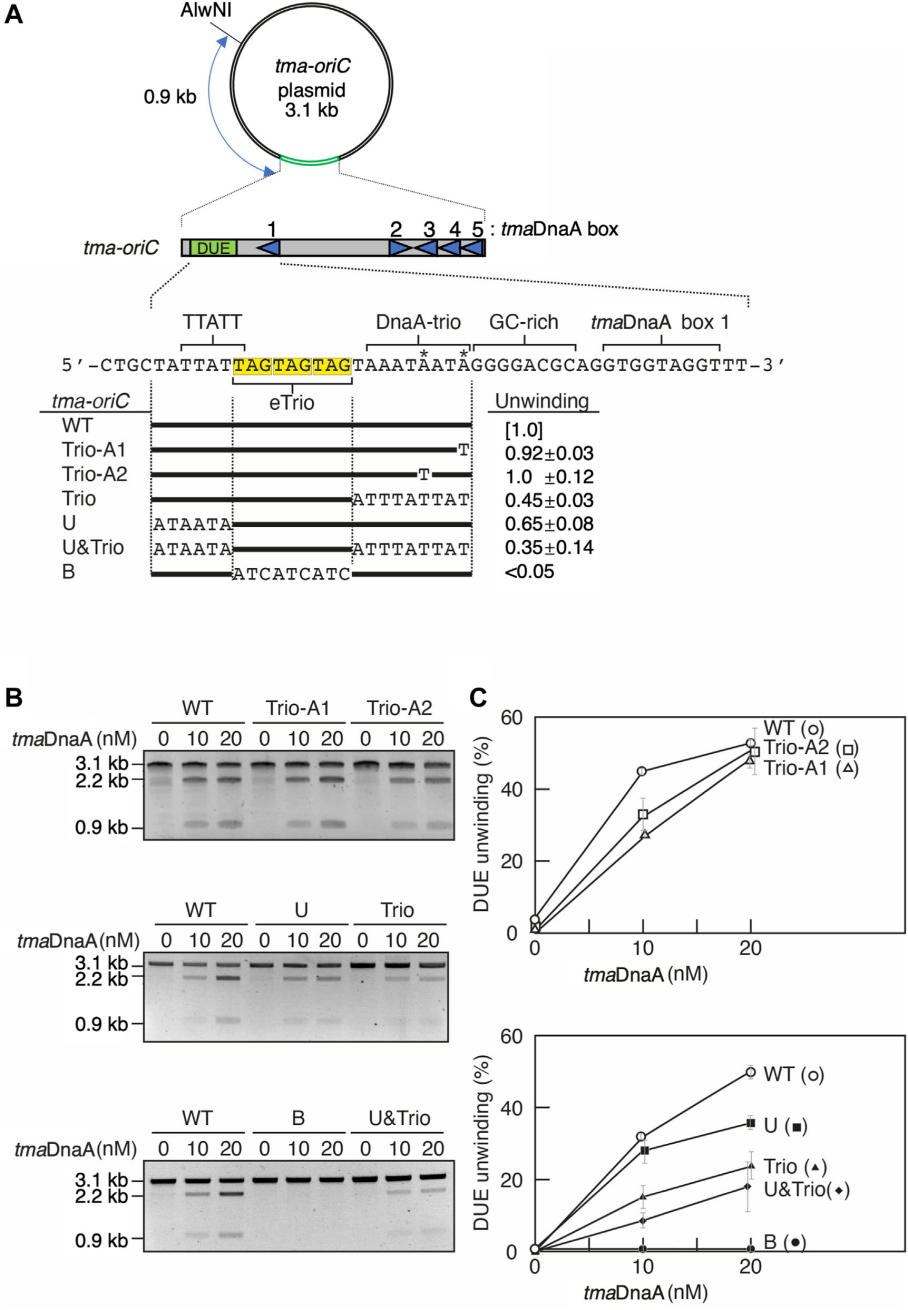
**The three tandem TAG repeats are essential for DUE unwinding**. *A*, the structure of *tma-oriC* plasmids. pOZ14 is a pBluescript derivative with a 149 bp minimal *tma-oriC* DNA (WT). *tma-oriC* is indicated by a *gray bar*, *tma*DUE by an *green box* (DUE), and *tma*DnaA boxes 1 to 5 by *blue triangles* (box 1–5). Sequences of the TTATT, eTrio, DnaA-trio, GC-rich, and *tma*DnaA box 1 motifs are shown below the *tma-oriC*, as are derivatives of the *tma*DUE region. For mutant *tma-oriC* plasmids, intact DUE regions are indicated by *bold lines* and base substitutions by *letters*. The percentages of the open complex at 20 nM *tma*DnaA (unwinding) are shown relative to that of the wildtype (WT). *B* and *C*, open complex formation. WT and mutant *tma-oriC* plasmids were individually incubated with the indicated concentrations of ATP-*tma*DnaA, followed by P1 nuclease digestion. After purification, DNA samples were further digested with AlwNI and analyzed by 1% agarose gel electrophoresis and ethidium bromide staining. (*B*) Gel images. (*C*) Mean ± standard deviation percentages of P1 nuclease-digested DNA (n = 2) quantified by FIJI software. DUE, duplex unwinding element.

The present study provides evidence showing that the *T. maritima* DUE consists of at least two functional modules, an unwinding module and a DnaA-binding module, and that ATP-*tma*DnaA head-to-tail oligomers can be constructed on a *tma*DnaA box-cluster with an inverted box. The *tma*DUE contained an *E. coli*-type TTATT motif and previously annotated DnaA-trios (5′-AAA-TAA-TA-3′) flanking *tma*DnaA box 1 (34) (Fig. 1*C*), with both stimulating unwinding without binding to *tma*DnaA–*tma*DOR complexes. By contrast, binding was strictly dependent on three direct repeats of the trinucleotide TAG located between the two elements. The present study also determined the optimal arrangement of the *tma*DnaA boxes, including their orientation and spacing. Notably, formation of head-to-tail oligomers of *tma*DnaA did not always require DnaA box clusters in the same direction. These findings provide insight into the DnaA oligomer-binding mechanisms in *oriC*s of many species that vary in the directions of DnaA boxes.

## Results

### Identification of a novel motif within *tma-oriC* required for DUE unwinding

To gain insights into prototypic structures of the origin, the origin sequences of the deep-branching hyperthermophilic bacterium, *T. maritima*, were analyzed. The 149 bp minimal *tma-oriC* in the *T. maritima* chromosome consisted of an AT-rich *tma*DUE and a flanking *tma*DOR containing the five *tma*DnaA boxes 1 to 5 recognized by *tma*DnaA (Fig. 2*A*). The 12-mer sequence motifs have been named *tma*DnaA boxes (40, 41). Based on further sequence analysis indicating that the terminal three bases of each motif are only moderately conserved (Fig. S2*B*) and previous data indicating that all *tma*DnaA boxes had similar affinity, irrespective of the variations in these three bases (40), the consensus sequence was redefined as the most conserved 9-mer, 5′-ACCTACCAC-3′, preserving its asymmetry. Moreover, the right part of *tma*DOR carrying *tma*DnaA boxes 3 to 5 was found to permit formation of distinct ATP-*tma*DnaA oligomers able to bind ss-*tma*DUE, most likely through the ssDUE recruitment mechanism (9). Examination of the *tma*DUE sequences suggested the importance of two ssDUE-binding motifs: TTATT from *E. coli* and the DnaA-trio from *B. subtilis* (Fig. 2*A*). Although each of these motifs has been implicated in initiation of replication of its respective organism, their functional importance in evolutionarily distant bacteria, such as *T. maritima*, was undetermined.

The DnaA-trios (5′-AAA-TAA-TA-3′) are previously annotated within *tma*DUE at the site proximal to *tma*DOR (34) (Fig. 2*A*). Importance of this sequence for open complex formation was assessed by P1 nuclease assays using *tma*DnaA, the *E. coli* DNA-binding protein HU, and a 3.1 kb supercoiled plasmid DNA containing the *tma-oriC* (Fig. 2*A*). HU protein is the evolutionarily highly conserved IHF homolog in eubacterial species and can sustain DUE unwinding of *E. coli oriC* instead of IHF which is conserved only in proteobacteria, nitrospirae, and nitrospinae (42, 43, 44, 45, 46). As we previously demonstrated (40), ATP-bound, but not ADP-bound, *tma*DnaA promotes open complex formation of *tma-oriC* at 48 °C in the presence of *E. coli* HU. In this assay, unwound *tma*DUE is detected by the endonuclease P1 promoting cleavage of the single-stranded DNA. Further digestion with the restriction enzyme AlwNI yields 2.2 and 0.9 kb DNA fragments (Fig. 2, *A*–*C*).

First, *tma*DUE unwinding was assessed using *tma-oriC* bearing mutations in the annotated DnaA-Trios (Fig. 2, *B* and *C*). Because a base substitution at either the first or third position from the 3′ end of the DnaA-trio sequence motif (5′-TAG-TAG-AAG-TAA-TAG-TA-3′, with the corresponding residues underlined), but not at the surrounding positions, was found to lead to severe initiation defects in *B. subtilis* (35), mutant *tma-oriC* plasmids bearing an A-to-T substitution at the corresponding positions (5′-AAA-TAA-TA-3′) were analyzed. The unwinding activities of these mutant plasmids were comparable to the activities of wildtype (WT) *tma-oriC* plasmid (Trio-A1 and Trio-A2 in Fig. 2, *B* and *C*). Moreover, moderate unwinding activities remained even when the entire DnaA-trio was scrambled (Trio in Fig. 2, *B* and *C*). Taken together, these results suggested that the previously annotated DnaA-trio plays a supportive, but not essential, role in open complex formation.

To further analyze the sequences essential for *tma*DUE unwinding, the TTATT motif was examined similarly (Fig. 2*A*). Scrambling of this sequence slightly inhibited *tma*DUE unwinding (U in Fig. 2, *B* and *C*). Moreover, when the TTATT and DnaA-trio sequences were simultaneously scrambled, the inhibition levels were additive, but slight unwinding activity remained (U&Trio in Fig. 2, *B* and *C*). These observations suggested that both the TTATT motif and the DnaA-trio are required for full *tma*DUE unwinding activity.

Analyses of the sequences located between the TTATT motif and the DnaA-trio showed three tandem repeats of TAG. Strikingly, scrambling of these sequences completely abolished the *tma*DUE unwinding activity (B in Fig. 2, *B* and *C*), indicating that these sequences were essential for open complex formation. Based on their homology to DnaA-trios, the three tandem TAG repeats have been named the extended Trio (eTrio).

### ATP-*tma*DnaA oligomers on DOR bind ssDUE through eTrio

**Figure 3:**
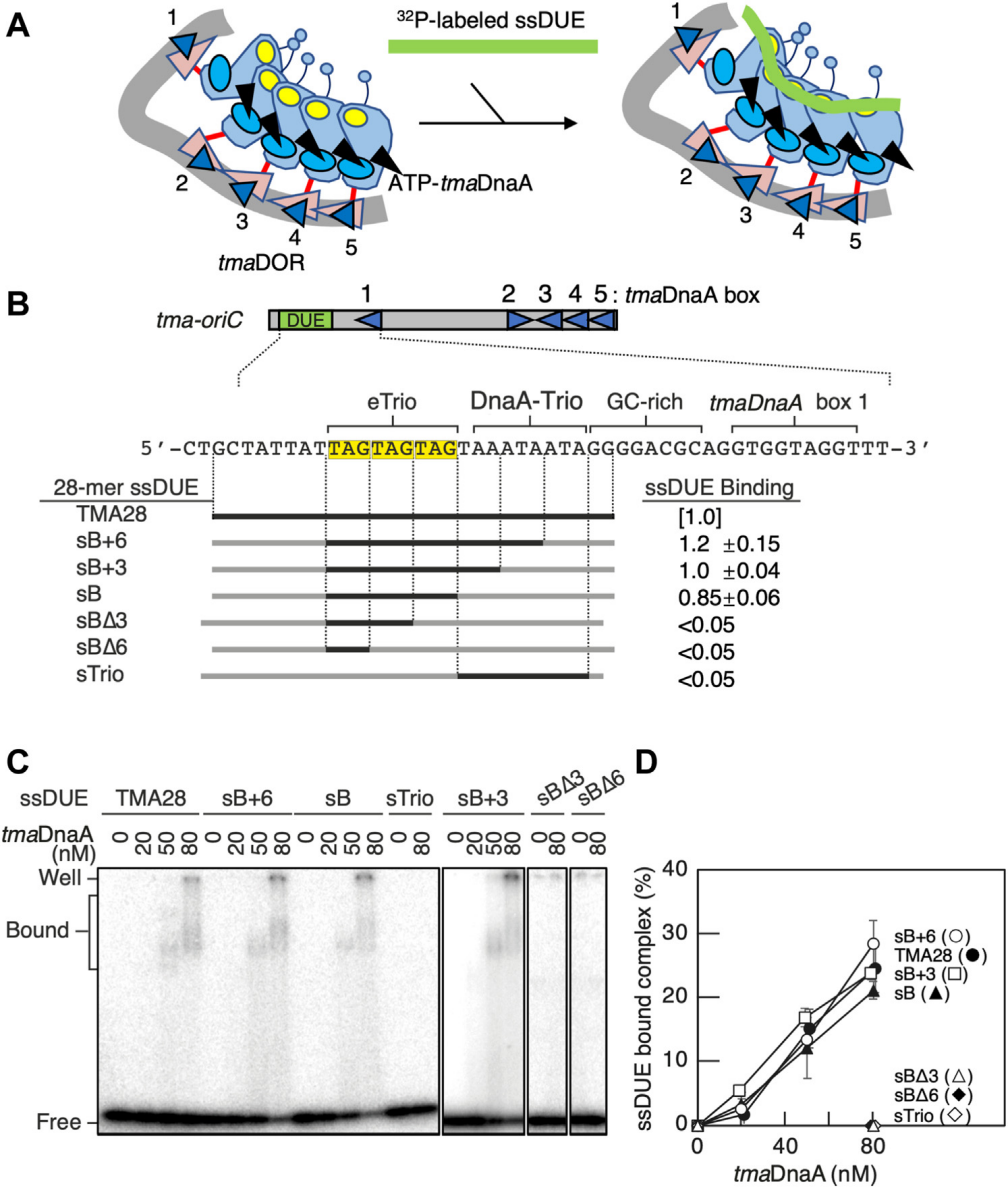
**The three tandem TAG repeats are essential for ssDUE binding**. *A*, schematic of the ssDUE-binding assay. ATP–*tma*DnaA–*tma*DOR complexes were constructed and incubated with ^32^P-labeled ssDUE, followed by EMSA. See the other data in this paper for overall structure of ATP–*tma*DnaA–*tma*DOR complexes. *B–D*, wildtype ssDUE fragment (TMA28) or its derivatives with oligo-dC-substitution (2.5 nM) were incubated with *tma*DOR (30 nM) and the indicated amount of ATP-*tma*DnaA, followed by EMSA. Minimal *tma-oriC* is shown as in Figure 2*A*. The ssDUE sequences used in this assay are illustrated schematically in *panel B*, with intact and oligo-dC-substituted sequences shown in *bold and gray lines*, respectively. *C*, representative gel images. *D*, quantitation of the results in (*C*) (n = 2) relative to the input ssDUE. Binding activities (ssDUE binding) at 80 nM *tma*DnaA are shown in *panel B* relative to that of TMA28. DOR, DnaA oligomerization region; EMSA, electrophoretic mobility shift assay; ssDUE, single-stranded duplex unwinding element; *tma*, *Thermotoga maritima.*

To further dissect the roles of sequence motifs within DUE, interactions between ss-*tma*DUE and ATP-*tma*DnaA oligomers constructed on *tma*DOR were analyzed by electrophoretic mobility shift assay (EMSA). In these assays, a radiolabeled, 28-mer ss-*tma*DUE probe was coincubated with ATP-*tma*DnaA and *tma*DOR, followed by polyacrylamide gel electrophoresis (Fig. 3*A*). As we previously reported (9), ATP-*tma*DnaA oligomers constructed on *tma*DOR (ATP–*tma*DnaA–*tma*DOR complexes) specifically interact with the ligand TMA28, an upper strand fragment of ss-*tma*DUE (Figs. 3*A* and S3). These interactions depend on *tma*DOR and ATP-bound, but not ADP-bound, *tma*DnaA (Fig. S3), as previously reported (9). DnaA proteins are apt to form irregular aggregates in the absence of DNA binding, remaining in the gel well. Moreover, a right part of *tma*DOR, including *tma*DnaA boxes 3 to 5, is sufficient to construct ATP–*tma*DnaA complexes capable of interacting with ss-*tma*DUE (9).

EMSAs using a set of ss-*tma*DUE variants with oligo-dC substitutions were performed to determine the minimal sequence required by the upper strand of ss-*tma*DUE to bind ATP–*tma*DnaA–*tma*DOR complexes. Because eTrio was found essential for DUE unwinding, its ability to bind ATP–*tma*DnaA–*tma*DOR complexes was analyzed, with results showing that the ss-*tma*DUE variant bearing the eTrio and oligo-dC regions (sB) displayed binding activity comparable to WT TMA28 or variants bearing eTrio and a partial DnaA-trio (sB+6 and sB+3) (Fig. 3, *B*–*D*). Moreover, all three TAG trinucleotides within eTrio were required for binding as reducing the number of TAG trinucleotides to two (sBΔ3) or one (sBΔ6) completely abolished the binding activity. Similarly, the ss-*tma*DUE variant bearing only the DnaA-trio and oligo-dC regions (sTrio) was inactive (Fig. 3, *B*–*D*), indicating that ss-*tma*DUE binding strongly depends on the three TAG repeats within eTrio. Taken together with the results showing that eTrio is strictly required for DUE unwinding (Fig. 2), these findings indicate that direct interactions between ss-eTrio and ATP–*tma*DnaA–*tma*DOR complexes are important in stabilizing open complexes. Because the DnaA-trio within *tma*DUE is dispensable for binding to ATP–*tma*DnaA–*tma*DOR complexes but plays only a supportive role in open complex formation, the DnaA-trio might assist in the process of initial AT-rich DNA-preferential duplex unwinding, reducing the stability of the duplex.

### Individual *tma*DnaA boxes within minimal *tma*DOR are crucial for unwinding

To investigate the mechanisms underlying *tma*DnaA-complex formation, the roles of individual *tma*DnaA boxes in DUE unwinding were analyzed. DNase I footprint analyses show binding of ATP-*tma*DnaA to *tma*DnaA boxes 1 to 5 within *tma*DOR (9), a finding supported by the EMSA results in the present study (Fig. S4). EMSA showed co-operative binding of ATP-*tma*DnaA molecules to *tma*DOR, as well as ADP-*tma*DnaA binding to *tma*DOR, the latter resulting from the absence of a competitor DNA and the occurrence of cage effects impeding diffusion, differing from the results of DNase I footprint experiments. In addition, deletion of *tma*DnaA box 5 from minimal *tma-oriC* is found to reduce the DUE unwinding activity to ∼50% of the intact sequence (40). Residual activity is completely abolished by the simultaneous deletion of *tma*DnaA boxes 4 and 5, suggesting that these *tma*DnaA boxes are essential for activity (40).

**Figure 4:**
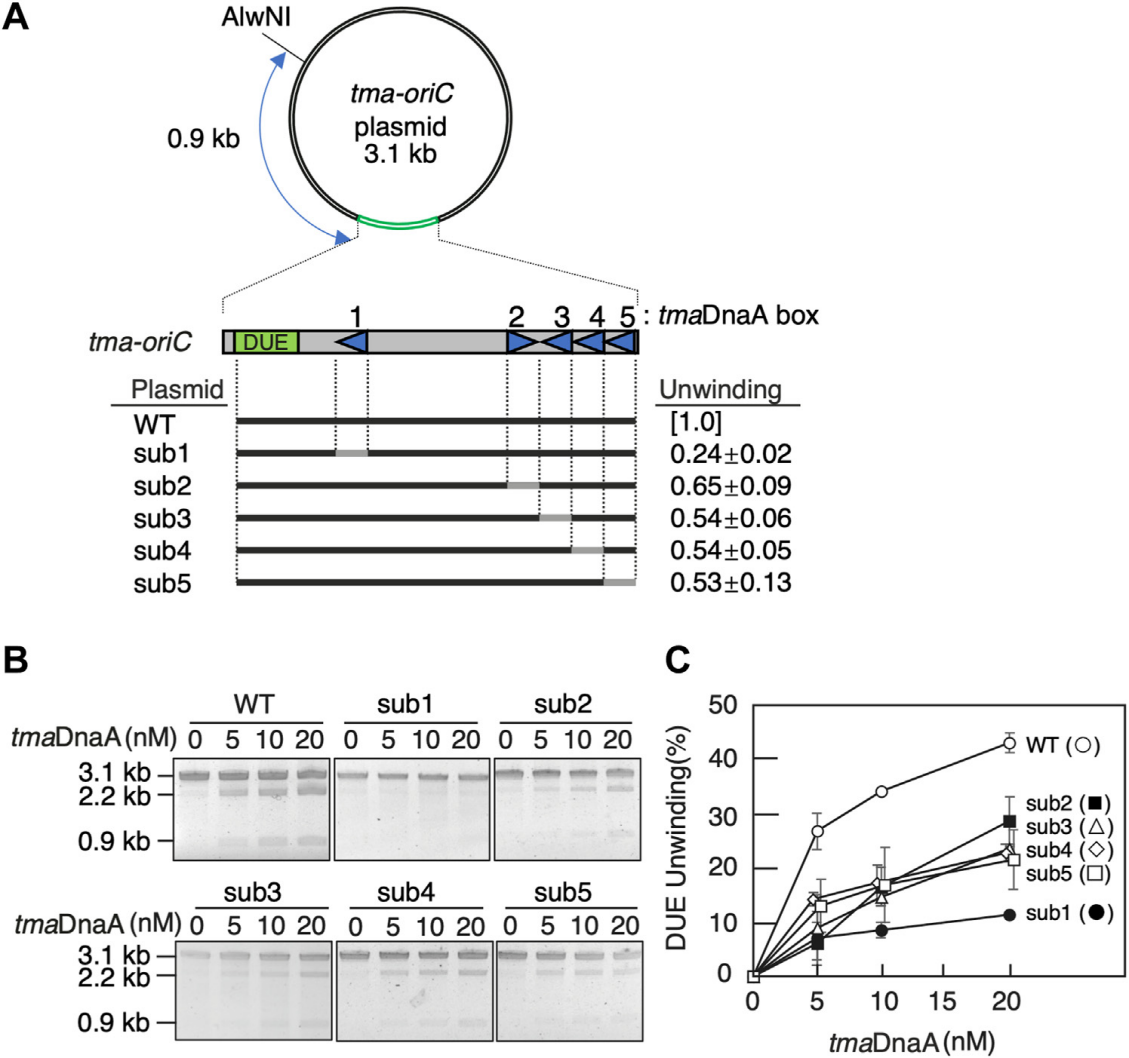
**Each of the *tma*DnaA boxes 1 to 5 within *tma*DOR is important for DUE unwinding**. Open complex formation by wildtype (WT) *tma-oriC* and its mutant derivatives (sub1-5), analyzed by P1 assays as described in the legend to Figure 2. *A*, schematic illustration of the *tma-oriC* plasmids. *Gray bars* indicate base substitutions, in which the 12-mer sequence, including the 9-mer *tma*DnaA box and its surrounding nucleotides, was replaced by the randomized sequence 5′-CCCAAGCAACAA-3’ (9). The WT sequence is indicated by *bold bars*. *B*, representative gel images. *C*, quantitative data (n = 2) shown as in Figure 2. The mean ± standard deviation activity relative to that of WT at a *tma*DnaA concentration of 20 nM are also shown. DOR, DnaA oligomerization region; DUE, duplex unwinding element; *tma*, *Thermotoga maritima*.

The requirements for individual *tma*DnaA boxes 1 to 5 were analyzed by performing P1 nuclease assays using a set of minimal *tma-oriC* plasmid pOZ14 derivatives in which each *tma*DnaA box sequence was randomized (Fig. 4*A*). Consistent with previous results, a mutation in *tma*DnaA box 4 or 5 each inhibited the unwinding activity by ∼50% (sub4 and sub5 in Fig. 4, *A*–*C*). Similarly, randomization of *tma*DnaA box 2 or 3 inhibited unwinding activity ∼50% (sub2 and sub3 in Fig. 4, *A*–*C*), and randomization of *tma*DnaA box 1 almost completely abolished unwinding activity (sub1 in Fig. 4, *A*–*C*). These results are consistent with the hypothesis that each of the five *tma*DnaA boxes plays a crucial role for full DUE unwinding activity. The stricter requirement of *tma*DnaA box 1 was also in good agreement with the ssDUE recruitment mechanism, in that ATP-*tma*DnaA bound to *tma*DnaA box 1 brings *tma*DUE close to ATP-*tma*DnaA complexes on the *tma*DnaA-complex–bound region (Fig. 3, *A* and *B*).

### Intact DNA helical turn between DUE and DOR is crucial for unwinding

**Figure 5:**
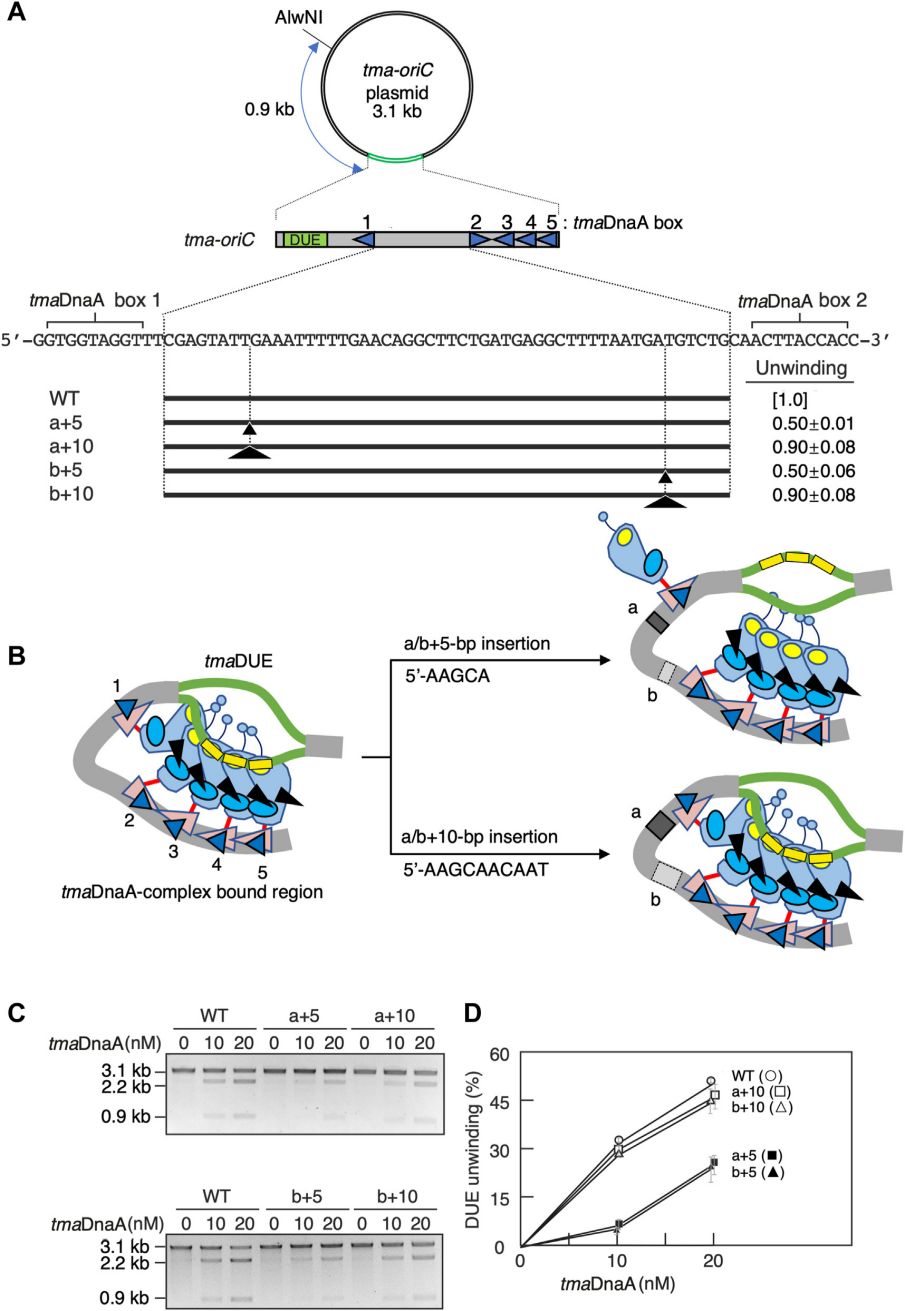
**The DNA helical turn between DUE and DOR underlies DUE unwinding**. *A*, the structure of the *tma-oriC* plasmids. Wildtype (WT) and mutant *tma-oriC* plasmids are shown as described in the legend to Figure 2. The positions of base insertions are indicated by small (+5) or large (+10) *arrowheads*. The amounts of the open complex at 20 nM *tma*DnaA (unwinding) are shown relative to that of WT. *B*, schematic presentation of the altered phasing between *tma*DUE and a *tma*DnaA-complex–bound region resulting from a 5 or 10 bp insertion within *tma*DOR. Presumable conformations of the complexes are shown based on the ssDUE recruitment mechanism. Inserted sequences are shown. *tma*DnaA and *tma*DnaA boxes are shown similar to that of *E. coli* DnaA in Figure 1 and the DnaAs in Figure 2, respectively. eTrio in *tma*DUE is indicated by *yellow rectangles*. *C* and *D*, open complex formation. WT and mutant *tma-oriC* plasmids were analyzed by P1 nuclease assays, as described in the legend to Figure 2. *C*, representative gel images and (*D*) quantitative data (n = 2) are shown as in Figure 2. DOR, DnaA oligomerization region; DUE, duplex unwinding element; eTrio, extended Trio; ss, single-stranded; *tma*, *Thermotoga maritima*.

To investigate the mechanisms required for open complex formation, importance of DNA helical turn differences in the *tma*DnaA box 1-box 2 intervening region in *tma*DUE unwinding was analyzed (Fig. 5*A*). Specifically, this study hypothesized that if the ssDUE recruitment mechanism was responsible for open complex formation, then inserting a full (10 bp) turn of a DNA helix would allow this region to retain its phasing, resulting in sustained unwinding activity (Fig. 5*B*). Conversely, inserting a half (5 bp) turn of a DNA helix would alter the phasing between the *tma*DUE-*tma*DnaA box 1 region and *tma*DnaA boxes 2 to 5, altering the interaction modes between the ATP-*tma*DnaA complexes and impairing interactions between the *tma*DUE and DnaA complexes.

These possibilities were tested by performing P1 nuclease assay using mutant *tma-oriC* plasmids with either a 5 bp or 10 bp insertion at a position flanking box 1 or box 2. DUE unwinding activity was fully preserved by insertion of a 10 bp fragment but was reduced by insertion of a 5 bp fragment (Fig. 5, *C* and *D*). These observations are in good agreement with the ssDUE recruitment mechanism, in that appropriate phasing between the DnaA complexes promotes these interactions *via* DNA looping, making this conformation crucial for the open complex at *tma-oriC*.

### Involvement of the Arg-finger in open complex formation

It was unclear whether a conventional AAA+-family-type head-to-tail interaction was involved in the formation of the open complex at *tma-oriC*. Evaluation of the *E. coli* DOR showed that the direct repeats of DnaA boxes reinforced formation of ATP-DnaA oligomers in a head-to-tail manner *via* interactions between the DnaA Arg285 Arg-finger and ATP bound to the adjacent protomer (14, 15) (Fig. 1*B*). Although the Arg-finger motif was found to be conserved at position 251 in *tma*DnaA, the minimal *tma-oriC* includes an inverted *tma*DnaA box 2. Thus, the mechanism by which the *tma*DnaA box 2-bound *tma*DnaA protomer interacts with the flanking *tma*DnaA protomers within the functional complex was unclear. Because previous DNase I footprint assays showed that ATP-*tma*DnaA, but not ADP-*tma*DnaA, binds co-operatively to the region from *tma*DnaA box 2 to box 5 (9), then the *tma*DnaA associated with the inverted *tma*DnaA box 2 should sustain its ability to interact with the adjacent *tma*DnaA molecule bound to *tma*DnaA box 3 through either conventional head-to-tail or nonconventional head-to-head interactions through the AAA+ domains.

**Figure 6:**
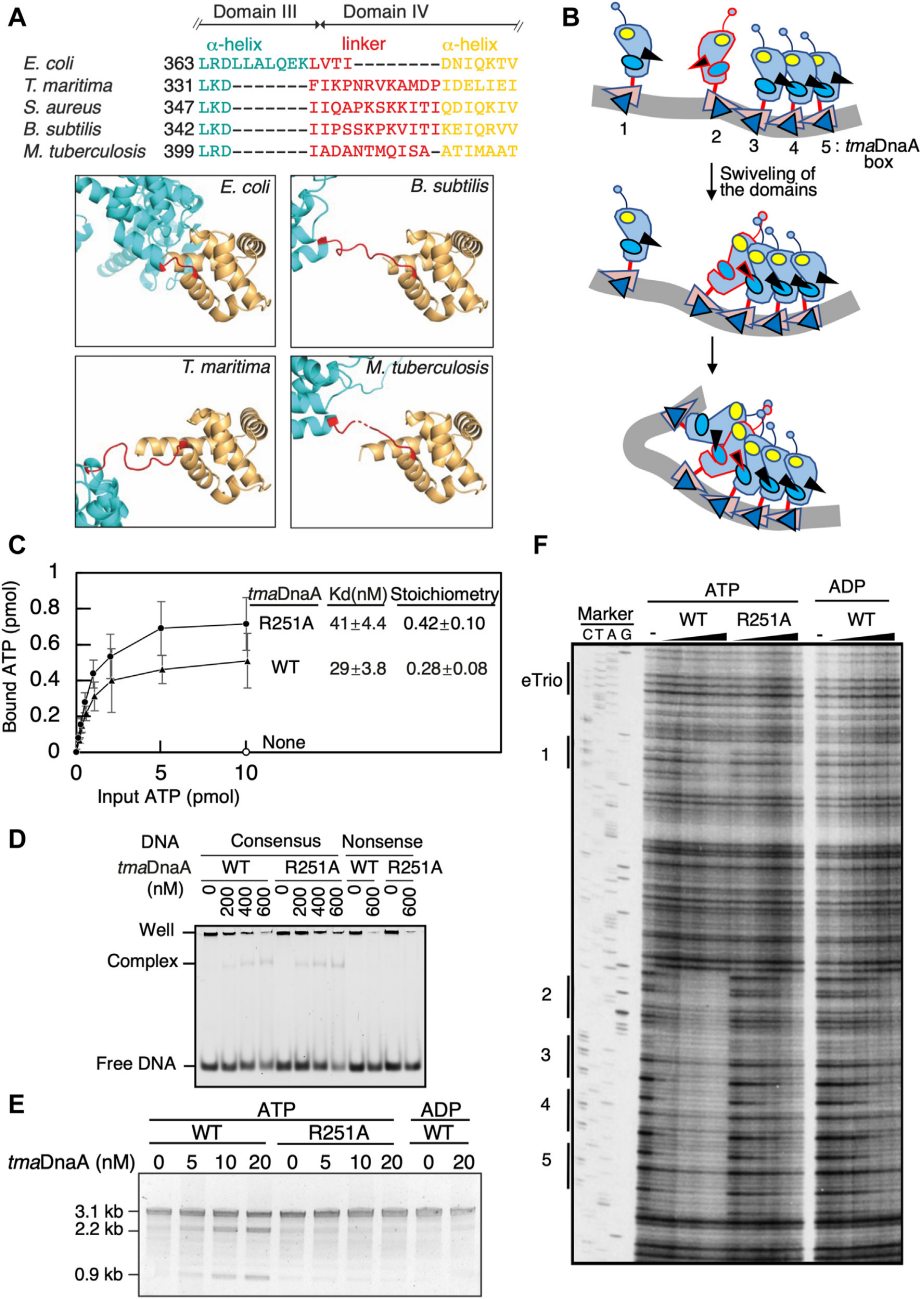
**The Arg251 Arg-finger of *tma*DnaA is required for open complex formation**. *A*, longer linkers between DnaA domains III and IV in DnaA orthologs than in *E. coli* DnaA. (*upper panel*) Alignment of the amino acid residues (*red letters*) forming the predicted linkers of *E. coli* DnaA and the indicated DnaA orthologs. (*lower panel*) DnaA structures predicted using AlfaFold2, with the predicted structures spanning the region from the C terminus of domain III to the N terminus of domain IV shown for comparison; linker regions are indicated in *red*. *B*, model for the Arg-finger–mediated oligomerization of the *tma*DnaA proteins on *tma*DnaA boxes 2 to 5. ATP-*tma*DnaA binds to each *tma*DnaA box, with the protomers on *tma*DnaA boxes 3 to 5 interacting in a head-to-tail manner through the function of the Arg-finger. Swiveling of the AAA+ domain of *tma*DnaA on box 2 allows the Arg-finger–mediated interaction of ATP-*tma*DnaA with the *tma*DnaA trimer on boxes 2 to 3, stabilizing the overall structure of the ATP-*tma*DnaA tetramer constructed on the region encompassing *tma*DnaA boxes 2 to 5. *C*, ATP-binding activity. Wildtype (WT) *tma*DnaA or *tma*DnaA R251 was mixed with various concentrations of radiolabeled ATP, followed by filter retention assays. Mean ± standard deviation activity (n = 3) are shown. The dissociation constant (Kd) and stoichiometry deduced from the Scatcherd plot are also indicted. *D*, DNA-binding activity. ADP-forms of WT *tma*DnaA or *tma*DnaA R251A were incubated with 18 bp DNA (300 nM) containing *tma*DnaA box 1 (5′-AGACCACCTACCACATAA-3’; *tma*DnaA box is underlined) or a nonsense DNA (5′-AGACCCAAGCAACAATAA-3′), followed by EMSA using an 8% polyacrylamide gel. *E*, open complex formation. The DUE unwinding activities of ATP-*tma*DnaA, ADP-*tma*DnaA, and ATP-*tma*DnaA R251 proteins were analyzed using pOZ14 DNA, as described in the legend to Figure 2. *F*, DNase I footprint. Various concentrations (0, 20, 40, 80, 150, 300, and 450 nM) of ATP-*tma*DnaA (ATP, WT), ADP-*tma*DnaA (ADP, WT), or ATP-*tma*DnaA R251A (ATP, R251A) were incubated for 10 min at 48 °C in buffer containing ^32^P-labeled *tma-oriC* DNA (10 nM), followed by DNA digestion with DNase I, 5% sequencing gel electrophoresis, and visualization using a BAS2500 Bio-imaging analyzer. The positions of DnaA boxes 1 to 5 and eTrio are determined by Sanger sequencing marker. AAA+, ATPases associated with diverse cellular activities; DUE, duplex unwinding element; EMSA, electrophoretic mobility shift assay; *tma, Thermotoga maritima*.

In *E. coli* DnaA, the C terminus of domain III (the AAA+ domain) is connected to the N terminus of domain IV (the DnaA box-binding domain) *via* a short flexible linker consisting of amino acids Leu367-Thr375, with the flexibility of this linker allowing limited swiveling of the two domains, as suggested by a study of the molecular dynamics underlying the construction of DnaA pentamers on left and right DORs (8). AlphaFold2-based structural model consistently suggested that *E. coli* DnaA contains a short flexible linker consisting of only four amino acid residues, Lue373-Ile376 (47). By contrast, similar modeling suggested that *tma*DnaA and other representative DnaA orthologs have corresponding linkers with a longer region consisting of 11 to 12 amino acid residues, allowing greater swiveling of the domains (Fig. 6*A*). Thus, a *tma*DnaA protomer bound to the inverted *tma*DnaA box 2 may use its flexible linker to revolve around its AAA+ domain, bringing the Arg-finger close to the ATP at the adjacent protomer bound to *tma*DnaA box 3 (Fig. 6*B*). The resultant head-to-tail interaction would stabilize the overall structure formed on *tma*DnaA boxes 2 to 5.

To confirm this hypothesis, the dependence of the co-operative ATP-*tma*DnaA interactions within the *tma*DnaA-complex–bound region on the Arg-finger motif was assessed. To investigate the specific role of this motif, a *tma*DnaA variant containing an Ala residue, rather than an Arg residue, at amino acid 251 was purified (R251A). A filter retention assay demonstrated that the affinity of *tma*DnaA R251A for ATP or ADP was comparable to that of WT *tma*DnaA (Figs. 6*C* and S5). Moreover, EMSA using a DNA carrying a single consensus *tma*DnaA box sequence demonstrated that the *tma*DnaA box-binding activity of *tma*DnaA R251A was similar to that of WT *tma*DnaA (Fig. 6*D*). Thus, the Arg-finger of *tma*DnaA is not required for binding to either ATP or a single *tma*DnaA box. By contrast, a P1 nuclease assay showed that *tma*DnaA R251A is virtually inactive for unwinding DUE, indicating that the Arg-finger is essential for open complex formation (Fig. 6*E*). These properties are fully consistent with those of the *E. coli* Arg-finger variant DnaA R285A (14).

DNase I footprint assays were subsequently performed to determine whether the Arg-finger was required for co-operative ATP-*tma*DnaA binding on the *tma-oriC*. The region encompassing *tma*DnaA boxes 2 to 5 was found to be readily protected against DNase I at low ATP-*tma*DnaA concentrations (*i.e.*, 20 nM), whereas much higher concentrations of ATP-*tma*DnaA (300–450 nM) were required for protection of *tma*DnaA box 1 (9) (Fig. 6*F*). Conversely, only high ADP-*tma*DnaA (300–450 nM) protected these *tma*DnaA boxes against DNase I. These profiles were fully consistent with our previous findings and with results indicating that ATP-*tma*DnaA, but not ADP-*tma*DnaA, binds co-operatively to the *tma*DnaA-complex–bound region. Notably, the footprint patterns of ATP-*tma*DnaA R251A were virtually indistinguishable from those of WT ADP-*tma*DnaA. These results indicated that co-operative ATP-*tma*DnaA binding to the region encompassing *tma*DnaA boxes 2 to 5 is strictly dependent on the R251 Arg-finger, supporting a model in which *tma*DnaA co-opts conventional head-to-tail interactions to form an open complex at *tma-oriC*, despite the involvement of the inverted *tma*DnaA box 2.

### Inverted *tma*DnaA boxes permit ATP-*tma*DnaA interactions in a head-to-tail manner

**Figure 7:**
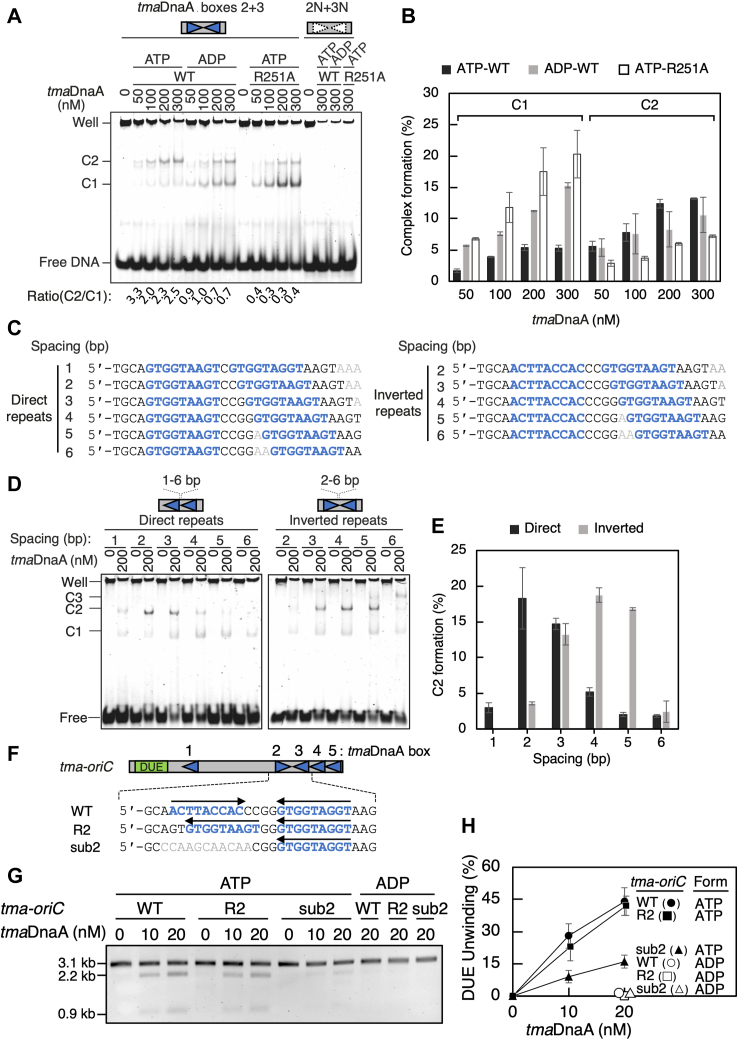
**Binding of *tma*DnaA on DNA with two tandem *tma*DnaA boxes**. *A* and *B*, ATP- or ADP-bound wildtype *tma*DnaA or ATP-bound *tma*DnaA R251A was incubated with a 30 bp DNA (300 nM) containing *tma*DnaA boxes 2 and 3 (*tma*DnaA boxes 2 and 3) or with a DNA fragment lacking *tma*DnaA boxes (2N and 3N). *A*, visualization of the *tma*DnaA–DNA complexes (C1 and C2) by 8% polyacrylamide gel electrophoresis and GelStar staining. *B*, percentages of the *tma*DnaA–DNA complexes (C1 and C2) relative to input DNA. *C–E*, ATP-*tma*DnaA (200 nM) was incubated with a 32 bp DNA (100 nM) containing direct or inverted repeats of *tma*DnaA boxes, with various spaces between boxes. *C*, sequences of the substrate DNA. *D*, visualization of the C2 complexes by 8% polyacrylamide gel electrophoresis and GelStar staining. *E*, percentages of C2 relative to input DNA. *F–H*, open complex formation. The P1 assay was performed as described in the legend to Figure 2. The *tma*-*oriC* mutant derivatives with inverted *tma*DnaA box 2 (R2) or randomized *tma*DnaA box 2 (Sub) are illustrated schematically (*F*). The gel image (*G*) and mean ± standard deviation percentages of P1 nuclease-digested DNA (n = 3) quantified by FIJI software (*H*) are shown respectively. Form, ATP- or ADP-forms of *tma*DnaA. *tma*, *Thermotoga maritima.*

To provide further evidence for this model, EMSA was performed using a DNA fragment with oppositely oriented *tma*DnaA boxes 2 and 3. Canonical head-to-tail interactions should stabilize the binding of the two *tma*DnaA molecules on DNA in a manner dependent on both ATP and the Arg-finger. Indeed, when WT ATP-*tma*DnaA was used, the predominant DNA complex contained two *tma*DnaA molecules (C2), whereas the complex containing one *tma*DnaA molecule (C1) was barely detected (Fig. 7, *A* and *B*). This preference for C2 formation was evident even at limited concentrations of ATP-*tma*DnaA (50, 100 nM). By contrast, C1 was the major product when WT ADP-*tma*DnaA or ATP-*tma*DnaA R251A was used. Thus, both ATP and the Arg-finger are involved in the efficient formation of C2 complexes. The residual amounts of C2 formed by WT ADP-*tma*DnaA and ATP-*tma*DnaA R251A suggest that subpopulations of these *tma*DnaA molecules could interact with each other in a manner dependent on neither ATP nor the Arg-finger: as observed for *E. coli* and *Streptomyces lividans* DnaAs (21, 23, 48, 49), weak domain I–domain I interaction and head-to-head domain III–domain III interaction might be involved.

The dependence of C2 formation on the spatial arrangement of the two repeated *tma*DnaA boxes was evaluated by EMSA using a set of direct or inverted repeats of *tma*DnaA boxes with different interspacing distance. The results suggested that formation of C2 depends on appropriate spacing between two *tma*DnaA boxes and their orientation (Fig. 7, *C*–*E*). Specifically, maximum C2 formation by two identically oriented *tma*DnaA boxes was observed when they were separated by 2 bp, as shown in the arrangements of *tma*DnaA boxes 3 to 4 and boxes 4 to 5. By contrast, maximum C2 formation by two inverted *tma*DnaA boxes was observed when they were separated by 4 bp interspace, as observed for *tma*DnaA boxes 2 to 3. Interestingly, a subpopulation of ATP-*tma*DnaA showed formation of a higher ordered complex C3 on the inverted *tma*DnaA repeat with separations of 5 or 6 bp (Fig. 7*D*). These findings suggest that there may be an as yet uncharacterized mode of inter-ATP–*tma*DnaA interactions on DNA. Moreover, the intervening space between two *tma*DnaA boxes and their orientation are key determinants for efficient formation of these dimeric complexes.

Finally, to confirm the concept that the inverted repeats of *tma*DnaA boxes allow for head-to-tail ATP–*tma*DnaA interactions to form an open complex at *tma-oriC*, a P1 nuclease assay was performed using a *tma-oriC* mutant derivative bearing the reversed *tma*DnaA box 2, aligning *tma*DnaA boxes 2 to 4 in the same direction with a 2-bp interspace (R2 in Fig. 7*F*). As a result, the unwinding activity of this mutant plasmid was comparable to that of the WT *tma-oriC* plasmid (Fig. 7, *G* and *H*). This finding contrasts with the compromised unwinding activity observed in the mutant plasmid with a randomized *tma*DnaA box 2 (Figs. 4 and 7, *G* and *H*). These observations suggest that *tma*DnaA box 2 is involved in open complex formation, regardless of its direction, highlighting a tunable nature of DnaA box orientation in the formation of bacterial initiation complexes at the origin.

## Discussion

**Figure 8:**
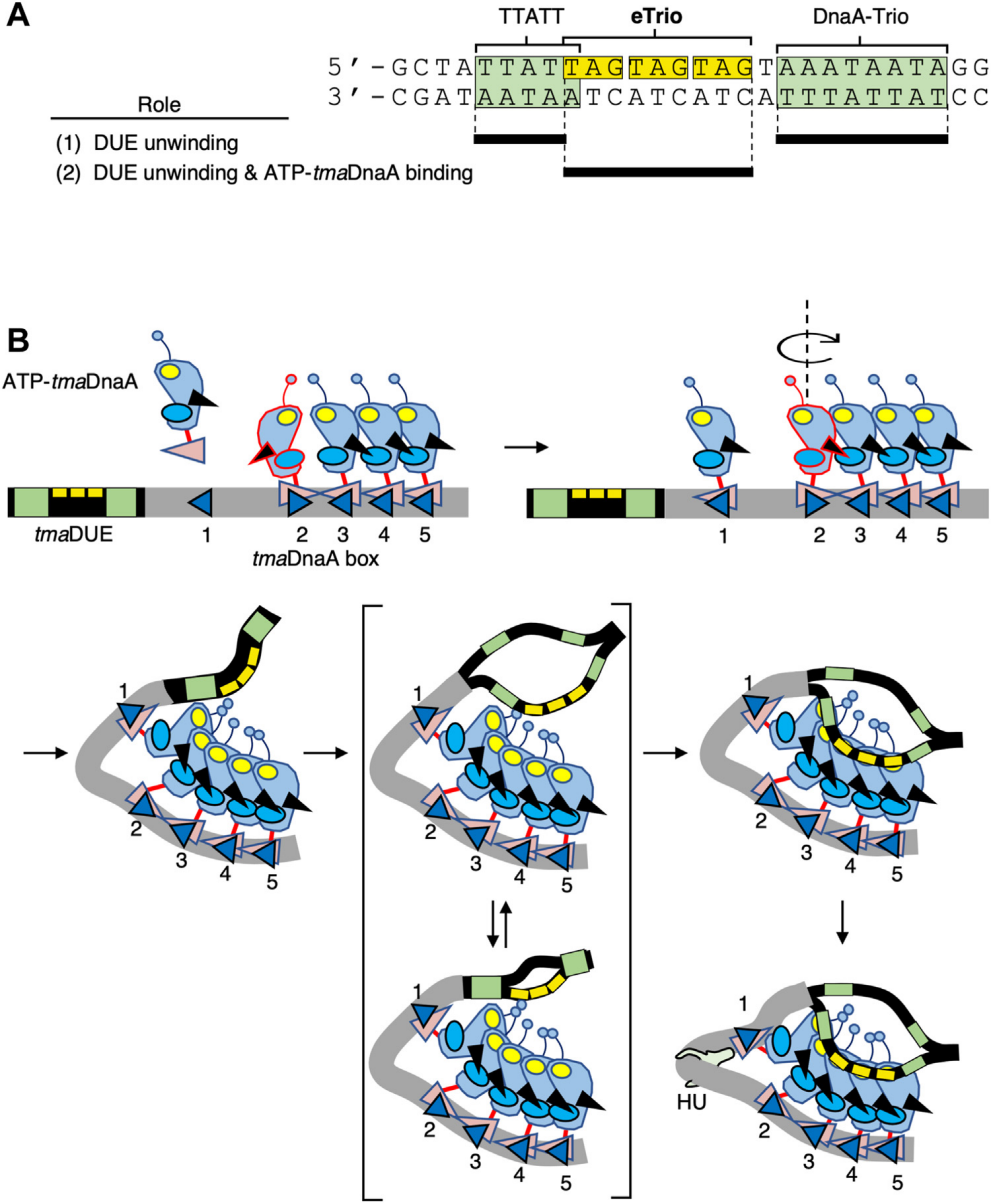
**Model for ssDUE recruitment mechanism in *T. maritima***. *A*, structure of *tma*DUE. The three tandem TAG repeats of eTrio are highlighted in *yellow*, and the TTATT motif and DnaA-trio are highlighted in *green*. Black bars indicate sites with crucial roles in DUE unwinding and/or ssDUE binding. *B*, model for open complex formation. The swiveled ATP-*tma*DnaA bound to *tma*DnaA box 2 facilitates formation of ATP-*tma*DnaA pentamers on *tma*DOR in a head-to-tail manner, inducing *tma*DUE unwinding. While TTATT and DnaA-trio engage in efficient DUE unwinding, the single-stranded eTrio directly binds to ATP-*tma*DnaA trimers bound to *tma*DnaA boxes 3 to 5, stabilizing the unwound state. A presumable HU binding to the spacer between *tma*DnaA boxes 1 and 2 further stabilizes open complex formation. DOR, DnaA oligomerization region; DUE, duplex unwinding element; eTrio, extended Trio; ss, single-stranded; *tma*, *Thermotoga maritima*.

In bacteria, despite the significant sequence homology among the DnaA family of proteins, the origins of bacterial genomes differ substantially in the number of DnaA boxes and their spatial arrangements. Moreover, the unwinding sequences have been insufficiently determined due to the lack of in-depth characterization using *in vitro* reconstituted systems. The present study analyzed *in vitro* reconstituted open complexes of the deep-branching hyperthermophilic bacterium, *T. maritima*, and identified eTrio consisting of three direct repeats of the trinucleotide TAG as a novel functional module within the unwinding sequences (Fig. 8*A*). These *in vitro* reconstituted systems using ATP-*tma*DnaA and *tma-oriC* provide concrete evidence that single-stranded eTrio interacts directly with ATP-*tma*DnaA oligomers constructed on *tma*DOR, thereby facilitating formation of the open complex. Moreover, the full activity of the open complex was dependent on the appropriate phasing between *tma*DUE-*tma*DnaA box 1 and *tma*DnaA boxes 2 to 5. These observations strongly suggest that ssDUE recruitment underlies the formation of the tripartite complex consisting of ATP-*tma*DnaA, *tma*DOR, and single-stranded eTrio (Fig. 8*B*). Because the right part of *tma*DOR, spanning *tma*DnaA boxes 3 to 5, is responsible for single-stranded DNA binding, and because a single DnaA molecule can be in direct contact with up to three nucleotides (9, 13), each *tma*DnaA box-bound ATP-*tma*DnaA protein on the right *tma*DOR likely senses a single TAG trinucleotide. *tma*DnaA box 2-bound *tma*DnaA might enhance this interaction by stimulating conformational changes of the *tma–oriC* complex (also see below).

Scrambling the sequences upstream and downstream of eTrio moderately inhibits duplex DNA unwinding. Although these regions have possible ssDUE-binding motifs, those have activities that are functionally distinct from those of eTrio in the stimulation of DUE unwinding. In papillomavirus, the adenine-thymine tracts flanked by the recognition sites of the initiator protein E2 are crucial for initiation of replication. Although these tracts are not in direct contact with the E2 protein, they display intrinsic DNA bending, thereby facilitating the ability of E2 proteins to recognize their binding sites (50). Similarly, the two AT-rich DNA regions flanking eTrio could contribute to local structural changes by destabilizing the duplex, thereby stimulating efficient unwinding of DUE and subsequent ssDUE binding to *tma*DnaA.

Taken together, these findings suggest a mechanism for open complex formation in *T. maritima* (Fig. 8*B*). ATP-*tma*DnaA proteins preferentially form a head-to-tail tetramer on *tma*DnaA boxes 2 to 5. The flexible nature of the linker between *tma*DnaA domains III and IV would allow considerable swiveling of the domains, whereby a *tma*DnaA protomer bound to the inverted *tma*DnaA box 2 swivels around its AAA+ domain to bring the Arg-finger close to the ATP on the adjacent protomer bound to *tma*DnaA box 3. When *tma*DnaA boxes 1 to 5 are all occupied by ATP-*tma*DnaA, the ATP-*tma*DnaA promoters bound to *tma*DnaA boxes 1 and 2 would interact transiently as a result of Brownian motion, inducing *tma*DUE unwinding. The unwound ssDUE would be stabilized though direct interaction of eTrio with the ATP-*tma*DnaA trimer bound to *tma*DnaA boxes 3 to 5. In addition, the interactions between the ATP-*tma*DnaA protomers bound to *tma*DnaA boxes 1 and 2 would induce bending of the DNA present in the space between *tma*DnaA boxes 1 and 2, stimulating ssDUE recruitment and stable unwinding. The site-specific HU binding to this space was recently exemplified using DMS footprint experiments (51). Given HU homologs are ubiquitous in the bacterial kingdom and HU-accessible interspaces between two DnaA boxes are basically present in the predicted bacterial origins (44, 51), it is conceivable that the HU-promoted ssDUE recruitment mechanism is prevailing among diverse bacterial species. Besides, the unwound region of tmaDUE may further expand over the TTATT and DnaA-trio motifs for stable unwinding and subsequent helicase loading. Expansion of the unwinding regions has been well characterized in open complexes of *E. coli* (7). This proposed mechanism is also fully consistent with the mechanism underlying ssDUE recruitment.

The observation that single-stranded eTrio is specifically recognized by the DOR-bound *tma*DnaA complexes also provides evolutionary insight into the functional structures required for eubacterial DUE. The DnaA-trios of *B. subtilis* and *H. pylori* that are crucial for single-stranded DNA binding of their cognate DnaA proteins contain multiple TAG trinucleotides like eTrio of *tma*DUE, suggesting that proteins in the DnaA family generally prefer single-stranded TAG repeats (34, 35). Consistently, the DnaA residues responsible for ssDUE binding are highly conserved in DnaA proteins (17). The molecular mechanisms by which *E. coli* DnaA and *tma*DnaA proteins recognize different ssDUE motifs remain to be elucidated in future.

Given the overall sequence dissimilarity of the AT-rich regions within the bacterial origins (Fig. S6), it is puzzling how evolutionarily distal bacteria such as *T. maritima*, *B. subtilis*, and *H. pylori* have co-opted TAG for initiation of replication. These bacteria thrive in different environments, leading us to speculate that the selective pressure for the DUE motifs may be determined by the chemical properties of DNA, rather than the environmental conditions. Supporting this hypothesis, the dinucleotide TA has shown flexible base pair morphology, including the ability to roll, tilt, and twist, thereby destabilizing the duplex structure (52, 53, 54). The DnaA proteins may therefore have evolved to bind to the transiently distorted TA, thereby stabilizing the unwound DNA. This is consistent with results showing that the TTATT motif with a central TA dinucleotide, instead of TAG, is present in *E. coli* DUE (7, 17). Intriguingly, TAG repeats have been observed in human telomeric DNA. Flexible TA dinucleotides contribute to pronounced DNA distortions necessary for formation of human telomeric nucleosome core particles (55).

The finding that inverted repeats of *tma*DnaA boxes can assist in head-to-tail interactions of ATP-*tma*DnaA protomers expands the view of tunability in the formation of bacterial initiation complexes at the origin. The predicted origins corresponding to the representative DnaA orthologs contain inverted DnaA boxes (Figs. 1 and S1). As in *tma*DnaA, the central AAA+ domain and the C-terminal DNA-binding domain in most, if not all, of those DnaA orthologs, are structurally predicted to be connected by flexible linkers containing 11 to 12 amino acid residues, which could engage in head-to-tail DnaA oligomerization on inverted repeats of DnaA boxes to form an initiation complex *via* the mechanism described in this study. Conversely, the array of direct DnaA box repeats at the *E. coli* origin represents a head-to-tail oligomerization of ATP-DnaA. Because *E. coli* DnaA carries a relatively short and presumably less flexible linker between the AAA+ and C-terminal DNA-binding domains, this linker likely coevolved with the architecture of the origin to maximize the efficiency of formation of the initiation complex. The *E. coli* chromosome also carries an inverted repeat of the DnaA box motifs within the DnaA-reactivating sequences 1 and 2, specific chromosomal loci for ADP-DnaA assembly that promote ATP-DnaA production by nucleotide exchange of ADP-DnaA (1, 56). This activity is facilitated by a head-to-head interaction of DnaA protomers on the inverted DnaA boxes (48). Thus, *E. coli* DnaA utilizes the structural constraint of the linker between the AAA+ and C-terminal DNA-binding domains to enhance regulatory systems on DnaA or to expand their repertoire.

In nature, the ssDUE recruitment mechanism has been co-opted, even by non-“DnaA-*oriC*” replication systems. This is exemplified by a recent study of *V. cholerae ori2* where the RctB initiator drives replication initiation (37). Similarly, the ssDUE recruitment mechanism could underlie initiation of the RK2 plasmid with the TrfA initiator (57). In either case, short oligonucleotide repeats are tandemly aligned in their corresponding DUEs, six direct repeats of ATCA in *V. cholerae ori2* DUE and four direct repeats of GGTT in RK2 plasmid DUE. These repeated sequences are likely favored by their cognate initiator proteins. Based on the mechanisms of action of different initiator proteins, these origins may have evolved independently from the DnaA-*oriC* system, thus expanding the mechanisms for ssDUE recruitment.

## Experimental procedures

### Strains and proteins

*E. coli* strain DH5α was used for cloning. *E. coli* HU, WT *tma*DnaA, and *tma*DnaA R251A were purified as described previously (40). Bovine serum albumin (BSA) was purchased from Roche and P1 nuclease from Wako.

### Buffers

Buffer E consisted of 60 mM Hepes-KOH (pH 7.6), 130 mM potassium glutamate, 7 mM EDTA, 7.5 mM dithiothreitol, 0.01% Triton X-100, 0.32 mg/ml BSA, 20% glycerol, and 3 μM ATP. Buffer P consisted of 60 mM Hepes-KOH (pH 7.6), 8 mM magnesium acetate, 0.1 mM zinc acetate, 30% glycerol, 0.32 mg/ml BSA, 100 mM potassium chloride, and 5 mM ATP. Buffer G consisted of 20 mM Hepes-KOH (pH 7.6), 1 mM EDTA (pH 8.0), 4 mM dithiothreitol, 5 mM magnesium acetate, 10% (v/v) glycerol, 0.1% Triton X-100, 0.1 mg/ml BSA, and 50 mM ammonium sulfate.

### DNA

The plasmids and oligonucleotides used in this study are listed in Tables 1 and 2, respectively.

**Table 1:** Plasmids used in this study

Plasmid	Description	Reference
pOZ14	A 3.1 kb pBluescript II derivative bearing a 149 bp minimal *tma-oriC*	(40)
pOZ14_Trio-A1	A pOZ14 derivative with modified DUE	This study
pOZ14_Trio-A2	A pOZ14 derivative with modified DUE	This study
pOZ14_U	A pOZ14 derivative with modified DUE	This study
pOZ14_B	A pOZ14 derivative with modified DUE	This study
pOZ14_Trio	A pOZ14 derivative with modified DUE	This study
pOZ14_U&Trio	A pOZ14 derivative with modified DUE	This study
pOZsub1	A pOZ14 derivative in which the sequence of *tma*DnaA box 1 is randomized	(9)
pOZsub2	A pOZ14 derivative in which the sequence of *tma*DnaA box 2 is randomized	(9)
pOZsub3	A pOZ14 derivative in which the sequence of *tma*DnaA box 3 is randomized	(9)
pOZsub4	A pOZ14 derivative in which the sequence of *tma*DnaA box 4 is randomized	(9)
pOZsub5	A pOZ14 derivative in which the sequence of *tma*DnaA box 5 is randomized	(9)
pOZ14_a+5	A pOZ14 derivative with a 5 bp insert between *tma*DnaA boxes 1 and 2	This study
pOZ14_a+10	A pOZ14 derivative with a 10 bp insert between *tma*DnaA boxes 1 and 2	This study
pOZ14_b+5	A pOZ14 derivative with a 5 bp insert between *tma*DnaA boxes 1 and 2	This study
pOZ14_b+10	A pOZ14 derivative with a 10 bp insert between *tma*DnaA boxes 1 and 2	This study
pOZ14_R2	A pOZ14 derivative in which the sequence of *tma*DnaA box 2 is reversed	This study
pTHMA-1	A plasmid for purification of wildtype *tma*DnaA	(40)
pTMA R251A	A pTHMA-1 derivative carrying the *tma*DnaA R251A allele	This study

**Table 2:** Oligonucleotides used in this study

Oligonucleotide	Sequence (5′-3′)	Reference
305_PAT3	GGGGACGCAGGTGGTAGGTTTC	(40)
306_PBS2	GTAATACGACTCACTATAGGGCGA	(40)
614_EMSA	CCCCCCCCCTAGCCCCCCCCCCCCCCCC	This study
615_EMSA	CCCCCCCCCTAGTAGCCCCCCCCCCCCC	This study
799_B	AGTGGATCCTGCTATTATATCATCATCTAAATAATAGGGGACGCAGGTGGTA	This study
803_U	AGTGGATCCTGCATAATATAGTAGTAGTAAATAATAGGGGACGCAGGTGGTA	This study
804_Trio	AGTGGATCCTGCTATTATTAGTAGTAGATTTATTATGGGGACGCAGGTGGTAGGTTTCGAG	This study
805_U&Trio	AGTGGATCCTGCATAATATAGTAGTAGATTTATTATGGGGACGCAGGTGGTAGGTTTCGAG	This study
823_PBS1	AATTAACCCTCACTAAAGGGAAC	(40)
824_b+5	ATCGAATTCAAACCTACCACTTACCTACCACTTACCTACCACCCGGGTGGTAAGTTGCAGACATGCTTTCATTAAAAGCCTCATCAGAAGCCT	This study
825_b+10	ATCGAATTCAAACCTACCACTTACCTACCACTTACCTACCACCCGGGTGGTAAGTTGCAGACAATTGTTGCTTTCATTAAAAGCCTCATCAGAAGCCT	This study
887_EMSA	CCCCCCCCTAGTAGTAGCCCCCCCCCCC	This study
888_EMSA	CCCCCCCCCCCCCCCCCTAAATAATACC	This study
889_EMSA	CCCCCCCCTAGTAGTAGTAACCCCCCCC	This study
890_EMSA	CCCCCCCCTAGTAGTAGTAAATACCCCC	This study
912_a+5	AGTGGATCCTGCTATTATTAGTAGTAGTAAATAATAGGGGACGCAGGTGGTAGGTTTCGAGTATTAAGCAGAAATTTTTGAACAGGCTTCTGATG	This study
913_a+10	AGTGGATCCTGCTATTATTAGTAGTAGTAAATAATAGGGGACGCAGGTGGTAGGTTTCGAGTATTAAGCAACAATGAAATTTTTGAACAGGCTTCTGATG	This study
929_Trio-A1	AGTGGATCCTGCTATTATTAGTAGTAGTAAATTATAGGGGACGCAGGTGGTAGGTTTC	This study
930_Trio-A2	AGTGGATCCTGCTATTATTAGTAGTAGTAAATAATTGGGGACGCAGGTGGTAGGTTTCGAG	This study
1031-Consense-1	AGAAAACCTACCACCTAA	(40)
1032-Consense-2	TTAGGTGGTAGGTTTTCT	(40)
1033-Nonsense-1	AGACCCAAGCAACAATAA	(40)
1034-Nonsense-2	TTATTGTTGCTTGGGTCT	(40)
1035-9mer-1	AGACCACCTACCACATAA	This study
1036-9mer-2	TTATGTGGTAGGTGGTCT	This study
1037-8mer-L-1	AGACCACCTACCAAATAA	This study
1038-8mer-L-2	TTATTTGGTAGGTGGTCT	This study
1039-8mer-R-1	AGACCCCCTACCACATAA	This study
1040-8mer-R-2	TTATGTGGTAGGGGGTCT	This study
1052-DnaABox2+3_1	TGCAACTTACCACCCGGGTGGTGGTAAAGT	This study
1053-DnaABox2+3_2	ACTTTACCACCACCCGGGTGGTAAGTTGCA	This study
1054-DnaABox2+3N_1	TGCAACTTACCACCCGGTGTTGTTGCAAGT	This study
1055-DnaABox2+3N_2	ACTTGCAACAACACCGGGTGGTAAGTTGCA	This study
1056-DnaABox2N+3N_1	TGCACAGGCAACACCGGTGTTGTTGCAAGT	This study
1057-DnaABox2N+3N_2	ACTTGCAACAACACCGGTGTTGCCTGTGCA	This study
1306_DnaABox 2+3(2)_1	TGCAACTTACCACCCGTGGTAGGTAAGTGGAA	This study
1307_DnaABox 2+3(2)_2	TTCCACTTACCTACCACGGGTGGTAAGTTGCA	This study
1308_DnaABox 2+3(3)_1	TGCAACTTACCACCCGGTGGTAGGTAAGTGGA	This study
1309_DnaABox 2+3(3)_2	TCCACTTACCTACCACCGGGTGGTAAGTTGCA	This study
1310_DnaABox 2+3(4)_1	TGCAACTTACCACCCGGGTGGTAGGTAAGTGG	This study
1311_DnaABox 2+3(4)_2	CCACTTACCTACCACCCGGGTGGTAAGTTGCA	This study
1316_DnaABox 2R+3(1)_1	TGCAGTGGTAAGTCGTGGTAGGTAAGTGGAAA	This study
1317_DnaABox 2R+3(1)_2	TTTCCACTTACCTACCACGACTTACCACTGCA	This study
1318_DnaABox 2R+3(2)_1	TGCAGTGGTAAGTCCGTGGTAGGTAAGTGGAA	This study
1319_DnaABox 2R+3(2)_2	TTCCACTTACCTACCACGGACTTACCACTGCA	This study
1320_DnaABox 2R+3(3)_1	TGCAGTGGTAAGTCCGGTGGTAGGTAAGTGGA	This study
1321_DnaABox 2R+3(3)_2	TCCACTTACCTACCACCGGACTTACCACTGCA	This study
1322_DnaABox 2R+3(4)_1	TGCAGTGGTAAGTCCGGGTGGTAGGTAAGTGG	This study
1323_DnaABox 2R+3(4)_2	CCACTTACCTACCACCCGGACTTACCACTGCA	This study
1338_2+3(+5)_1	TGCAACTTACCACCCAGGGTGGTAGGTAAGTG	This study
1339_2+3(+5)_2	CACTTACCTACCACCCTGGGTGGTAAGTTGCA	This study
1340_2+3(+6)_1	TGCAACTTACCACCCAAGGGTGGTAGGTAAGT	This study
1341_2+3(+6)_2	ACTTACCTACCACCCTTGGGTGGTAAGTTGCA	This study
1342_2R+3(+5)_1	TGCAGTGGTAAGTCCAGGGTGGTAGGTAAGTG	This study
1343_2R+3(+5)_2	CACTTACCTACCACCCTGGACTTACCACTGCA	This study
1344_2R+3(+6)_1	TGCAGTGGTAAGTCCAAGGGTGGTAGGTAAGT	This study
1345_2R+3(+6)_2	ACTTACCTACCACCCTTGGACTTACCACTGCA	This study
1594_2R_R	ATATCGAATTCAAACCTACCACTTACCTACCACTTACCTACCACCCACTTACCACACTGCAGAC	This study
TMA28	GCTATTATTAGTAGTAGTAAATAATAGG	(9)

To construct pOZ14_Trio-A1, pOZ14_Trio-A12, pOZ14_a+5, pOZ14_a+10, pOZ14_b+5, pOZ14_b+10, pOZ14_U, pOZ14_B, pOZ14_Trio, pOZ14_U&Trio, and pOZ14_R2, the inserted DNA fragments were amplified by PCR using pOZ14 and mutagenic primers (929/306 for pOZ14_Trio-A1, 930/306 for pOZ14_Trio-A2, 912/306 for pOZ14_a+5, 913/306 for pOZ14_a+10, 824/823 for pOZ14_b+5, 825/823 for pOZ14_b+10, 799/306 for pOZ14_B, 803/306 for pOZ14_U, 804/306 for pOZ14_Trio, 805/306 for pOZ14_U&Trio, and 1594/823 for pOZ14_R2). The amplified products were digested with BamHI and EcoRI and ligated into the BamHI and EcoRI sites of pBluescript II (Stratagene).

To construct pTMA R251A, the mutation was introduced into pTHMA-1 using a QuikChange site-directed mutagenesis kit, according to the manufacturer’s instructions (Stratagene) (16, 17).

### P1 nuclease assay

P1 nuclease assays were performed essentially as described (17, 40). Briefly, ATP-*tma*DnaA (0–20 nM) and pOZ14 or its mutant derivatives (400 ng; 4 nM) were incubated in buffer P (50 μl) containing *E. coli* HU (16 ng; 17 nM) for 10 min at 48 °C, followed by digestion with 1.5 units P1 nuclease for 200 s at 48 °C. The DNA samples were extracted with phenol/chloroform, precipitated with ethanol, and further digested with the restriction enzyme AlwNI. The products were analyzed using 1% agarose gel electrophoresis and ethidium bromide straining.

P1 assays described in Figure 7, *G* and *H* were performed using buffer P containing 40 mM ammonium sulfate, instead of 100 mM potassium chloride, which did not affect the specific nuclease activity.

### Electrophoretic mobility shift assay

EMSAs were performed essentially as described (7, 9). Briefly, ATP-*tma*DnaA (0–80 nM) and a 203 bp DNA fragment–containing *tma*DOR (30 nM) were incubated for 5 min at 48 °C, followed by electrophoresis on 4% polyacrylamide gels and visualization of *tma*DOR using GelStar dye (Lonza). To analyze the formation of ss-*tma*DUE–ATP–*tma*DnaA–*tma*DOR complexes, ATP-*tma*DnaA (0–80 nM) and the 203 bp DNA fragment containing *tma*DOR (30 nM) were incubated for 5 min at 48 °C; following the addition of ^32^P-labeled ss-*tma*DUE (2.5 nM), the reaction mixtures were incubated for an additional 5 min at 48 °C. The mixtures were subjected to 4% polyacrylamide gel electrophoresis, with *tma*DOR and ^32^P-labeled ss-*tma*DUE visualized using GelStar dye and Typhoon FLA 9500, respectively.

### DNase I footprint experiments

DNase I footprint experiments were performed essentially as described (9). Briefly, a ^32^P-end-labeled *tma-oriC* fragment (303 bp) was prepared by PCR using pOZ14 DNA and the primers 823 and ^32^P-end-labeled 306. The labeled DNA (10 nM) was incubated with WT *tma*DnaA or *tma*DnaA R251A (0–450 nM) for 10 min at 48 °C in buffer G (10 μl) containing 5 mM calcium acetate and 3 mM ATP or ADP, followed by incubation with DNase I (0.83 mU) for 4 min at the same temperature. DNA samples were analyzed by sequencing gel electrophoresis.

### Filter retention assay

Filter retention assays were performed as described previously (40). Briefly, WT *tma*DnaA or *tma*DnaA R251A (1.9 pmol) was preincubated for 5 min at 38 °C in buffer (25 μl) containing 50 mM Hepes-KOH (pH 7.6), 0.3 mM EDTA, 7 mM dithiothreitol, 20% glycerol, 0.007% Triton X-100, and [α-^32^P] ATP or [^3^H] ADP. Following addition of 5 mM magnesium acetate, the samples were further incubated on ice for 15 min and filtered through nitrocellulose membranes. The retained radioactivity was quantified using a liquid scintillation counter.

## Data availability

All data are available in the article.

## Supporting information

This article contains supporting information (32).

## Conflict of interest

The authors declare that they have no conflicts of interest with the contents of this article.
