# Supplementary material for: Thermotoga maritima oriC involves a DNA unwinding element with distinct modules and a DnaA-oligomerizing region with a novel directional binding mode
Source: J Biol Chem. 2023 Jun 3;299(7):104888. doi: 10.1016/j.jbc.2023.104888 (PMC10316083; doi:10.1016/j.jbc.2023.104888)
Supplement: Supporting information [file mmc1.docx]

**Supporting Information**

**
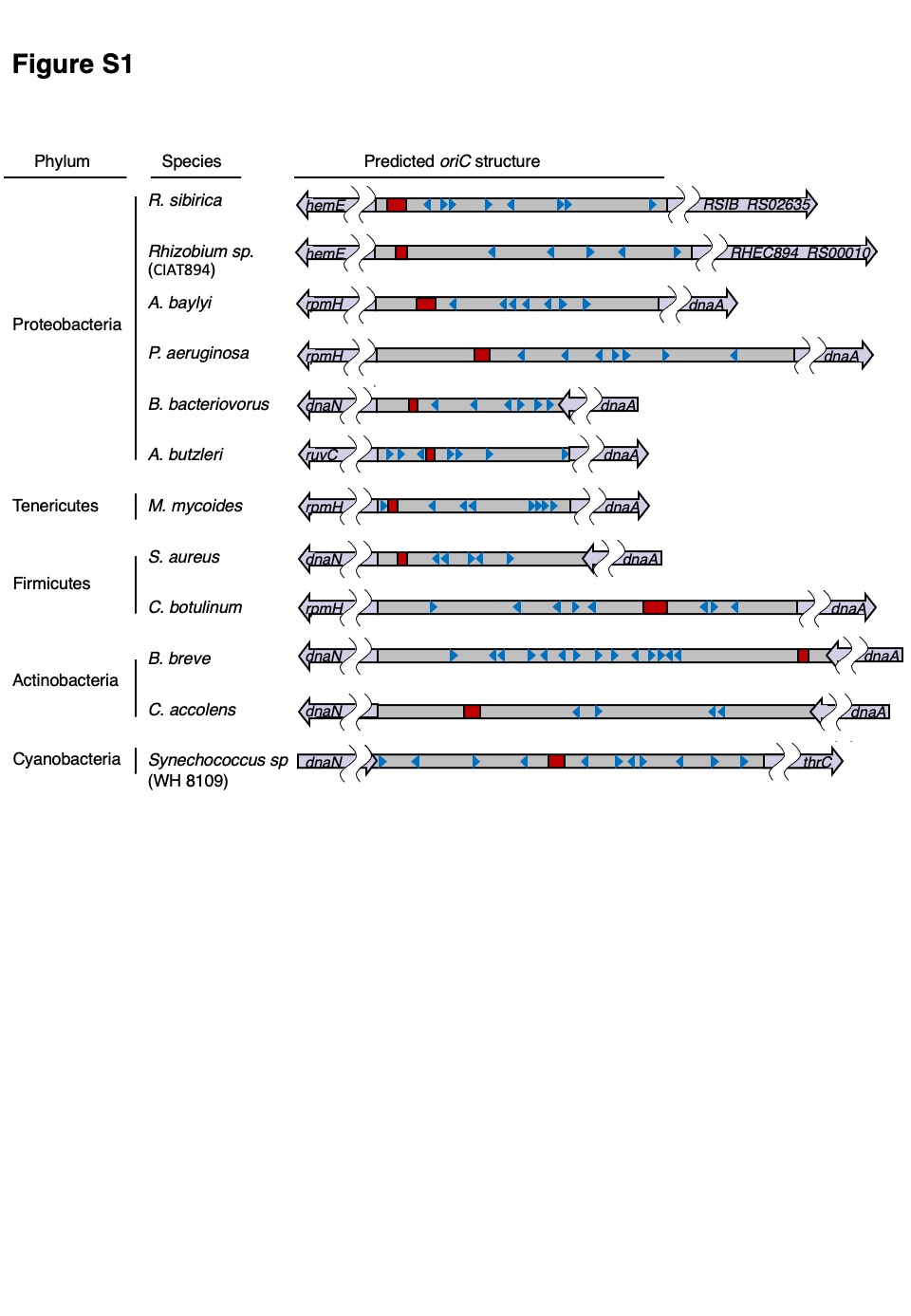
**

**Figure S1 Predicted eubacterial *oriC* structures**

Comparison of bioinformatically predicted origin sequences from various eubacterial species. Sequence information, including DnaA boxes (triangles) and AT-rich sequence motifs (red boxes), was obtained from the D*oriC* database (Dong *et al*., 2023). Arbitrarily selected origins are shown. Abbreviations: *R. sibirica, Rickettsia sibirica; A. baylyi, Acinetobacter baylyi; P. aeruginosa, Pseudomonas aeruginosa; B, bacteriovorus; A. butzleri, Arcobacter butzleri; M. mycoides, Mycoplasma mycoides; S. aureus, Staphylococcus aureus; C. botulinum, Clostridium botulinum; B. breve, Bifidobacterium breve; C. accolens, Corynebacterium accolens.*

**
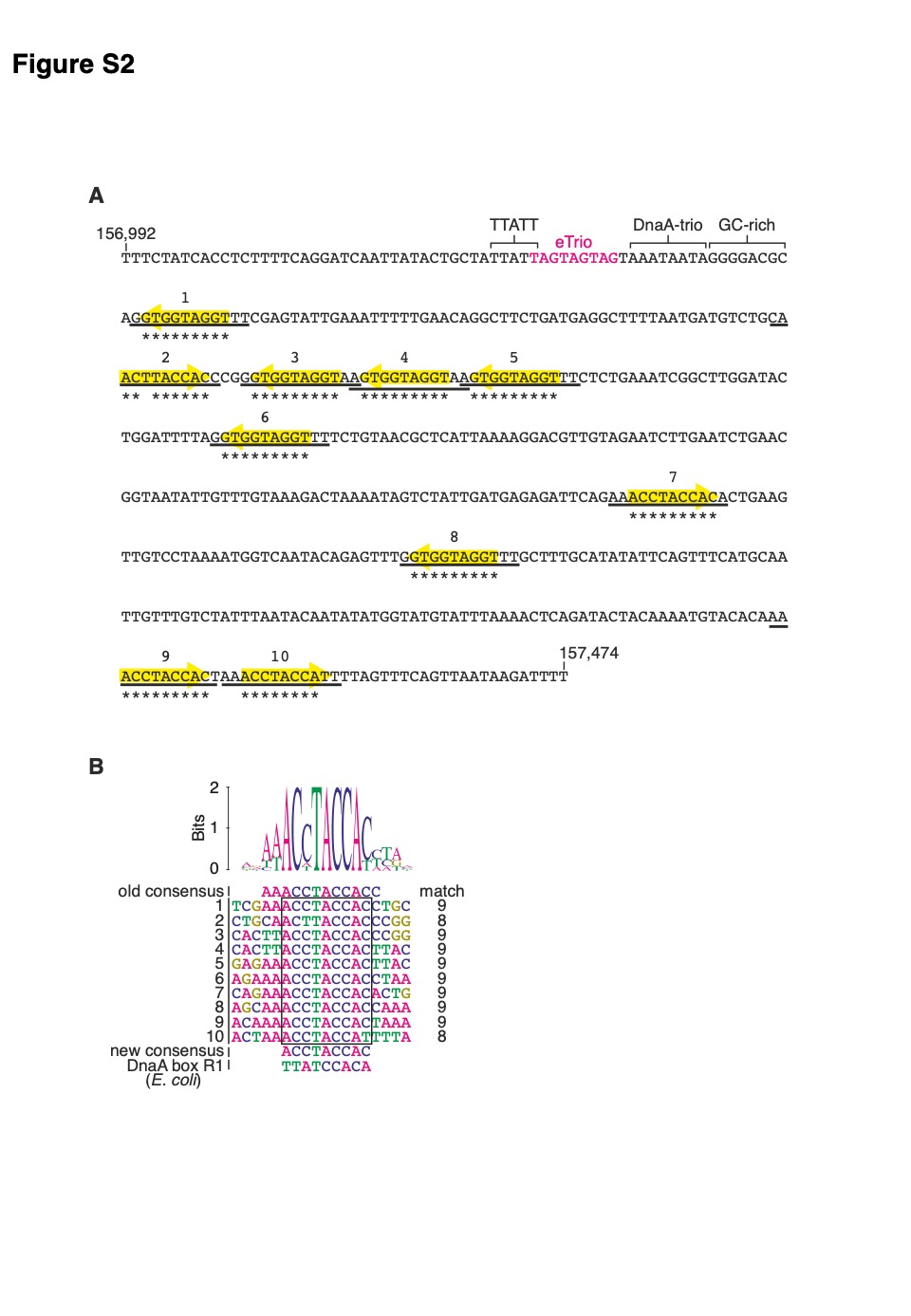
 Figure S2 The structure of *tma-oriC***

(A) The nucleotide sequence of *tma-oriC* (nt 156,992-157,474 from the *T. maritima* MSB8 genome) is shown, along with previously annotated *tma*DnaA boxes 1–10 (underlined), TTATT, DnaA-trio, and GC-rich motifs. Newly defined *tma*DnaA boxes and the motif orientation are indicated by yellow arrows. Sequences identical to the consensus *tma*DnaA box motif are highlighted by asterisks.

(B) Sequence alignment and logo for *tma*DnaA boxes. For comparison, the *E. coli* DnaA box R1 sequence is also shown.

**
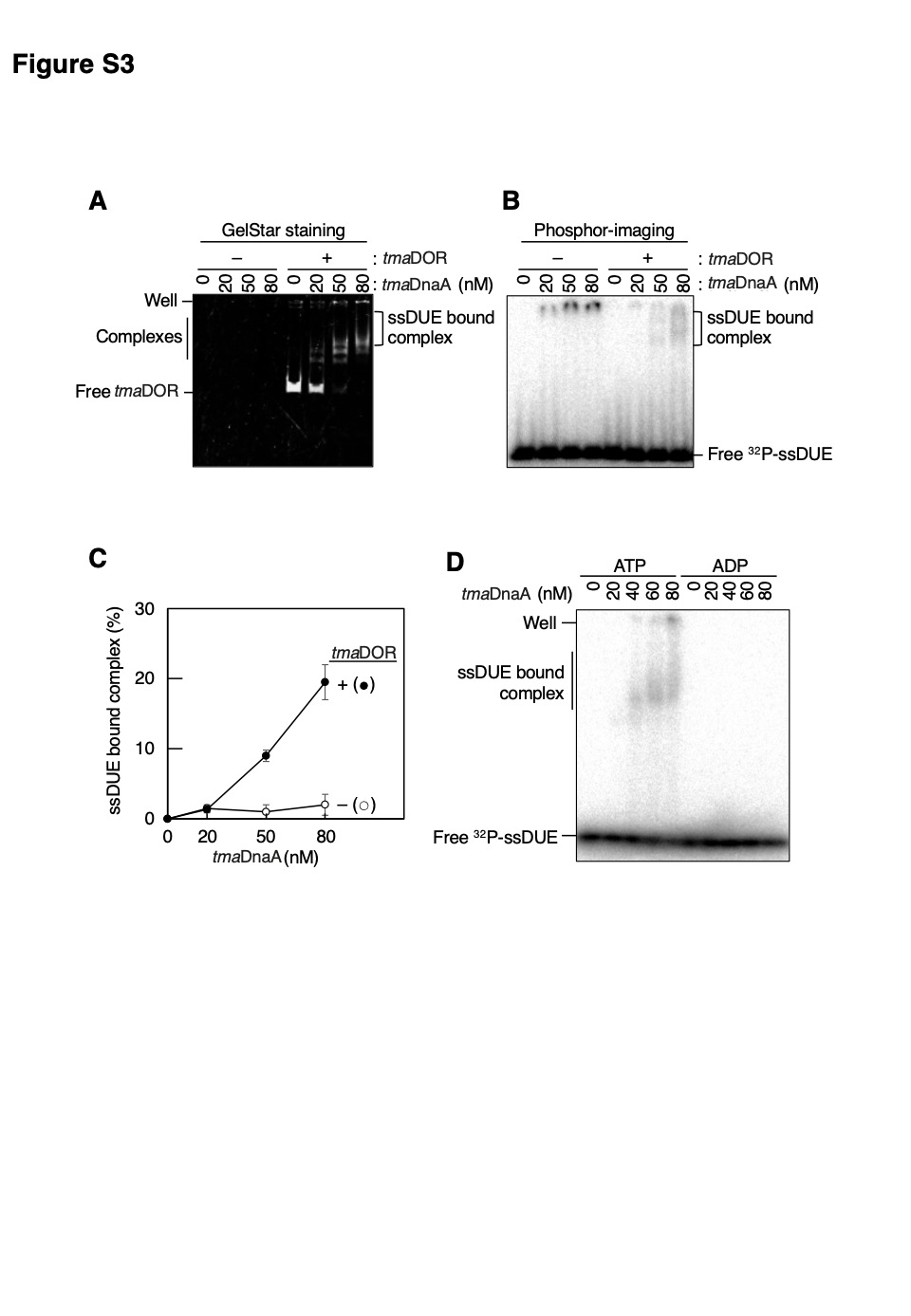
Figure S3 ss-*tma*DUE interactions with ATP-*tma*DnaA-*tma*DOR complexes**

(A-C) ATP-*tma*DnaA was incubated with (+) or without (-) *tma*DOR (15 nM) and further incubated with wild-type (WT) ^32^P-ss-*tma*DUE (1.5 nM), followed by EMSA using a 4% polyacrylamide gel. (A) Visualization of dsDNA by GelStar staining. (B) Determination of ^32^P-ss-*tma*DUE by phosphor-imaging. (C) Quantitation of the amounts of ^32^P-ss-*tma*DUE bound to ATP-*tma*DnaA-*tma*DOR complexes in (B), with the percentages relative to input *Tma*-ssDUE plotted as ssDUE bound complexes. Abbreviations: Free, protein-free DNA; Complexes, ATP-*tma*DnaA-*tma*DOR complexes; Well, gel well; ssDUE bound complex, ATP-*tma*DnaA-*tma*DOR-^32^P-ss-*tma*DUE complex.

(D) Phosphor-imaging for titration of ATP/ADP-*tma*DnaA in the presence of *tma*DOR and WT ^32^P-ss-*tma*DUE.

**
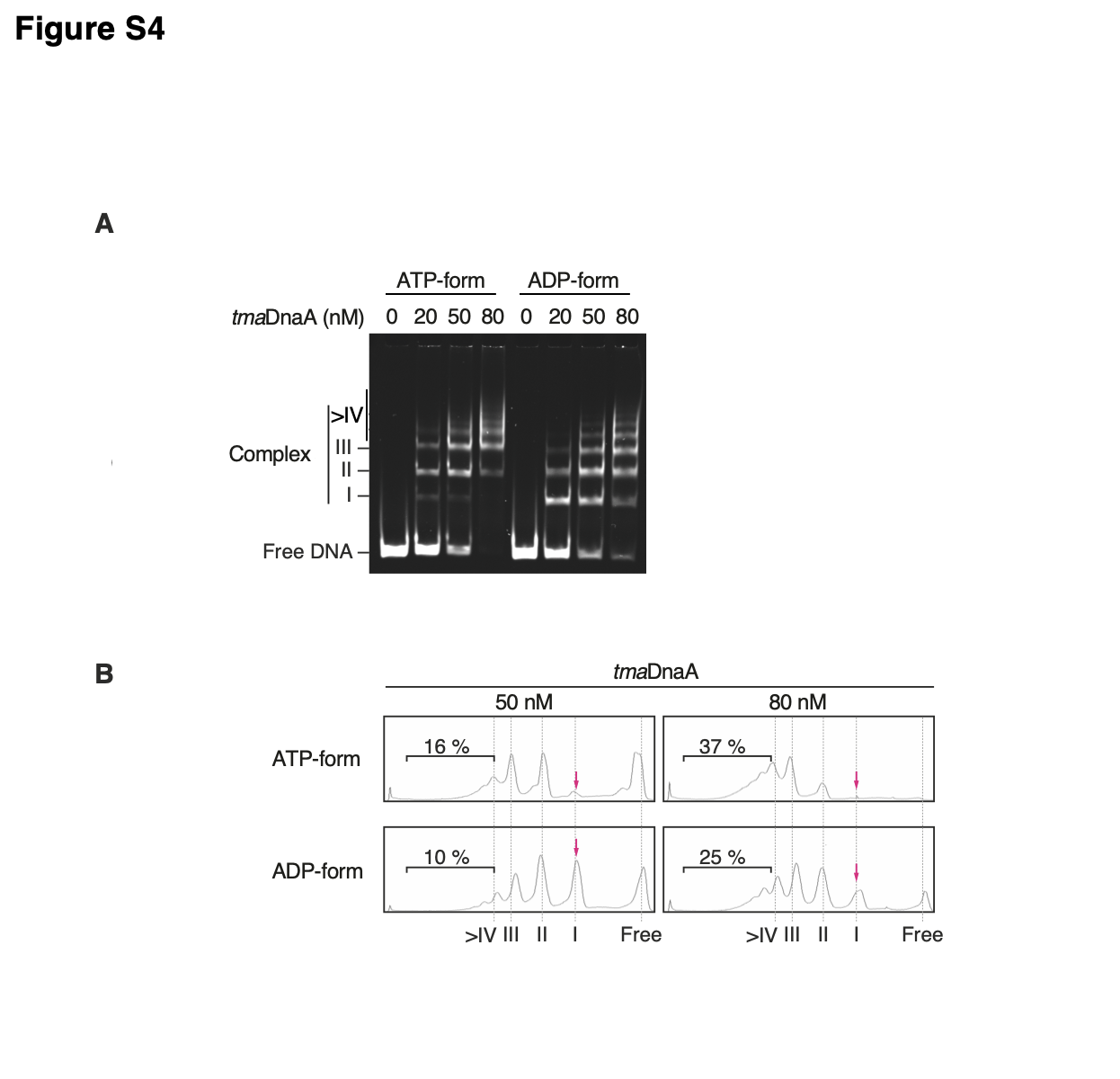
**

**Figure S4 ATP-*tma*DnaA binding of *tma*DOR**

A 203 bp dsDNA (30 nM) containing wild-type *tma*DOR was incubated with the indicated concentrations of ATP-*tma*DnaA or ADP-*tma*DnaA, followed by EMSA. DNA bands were visualized by GelStar staining.


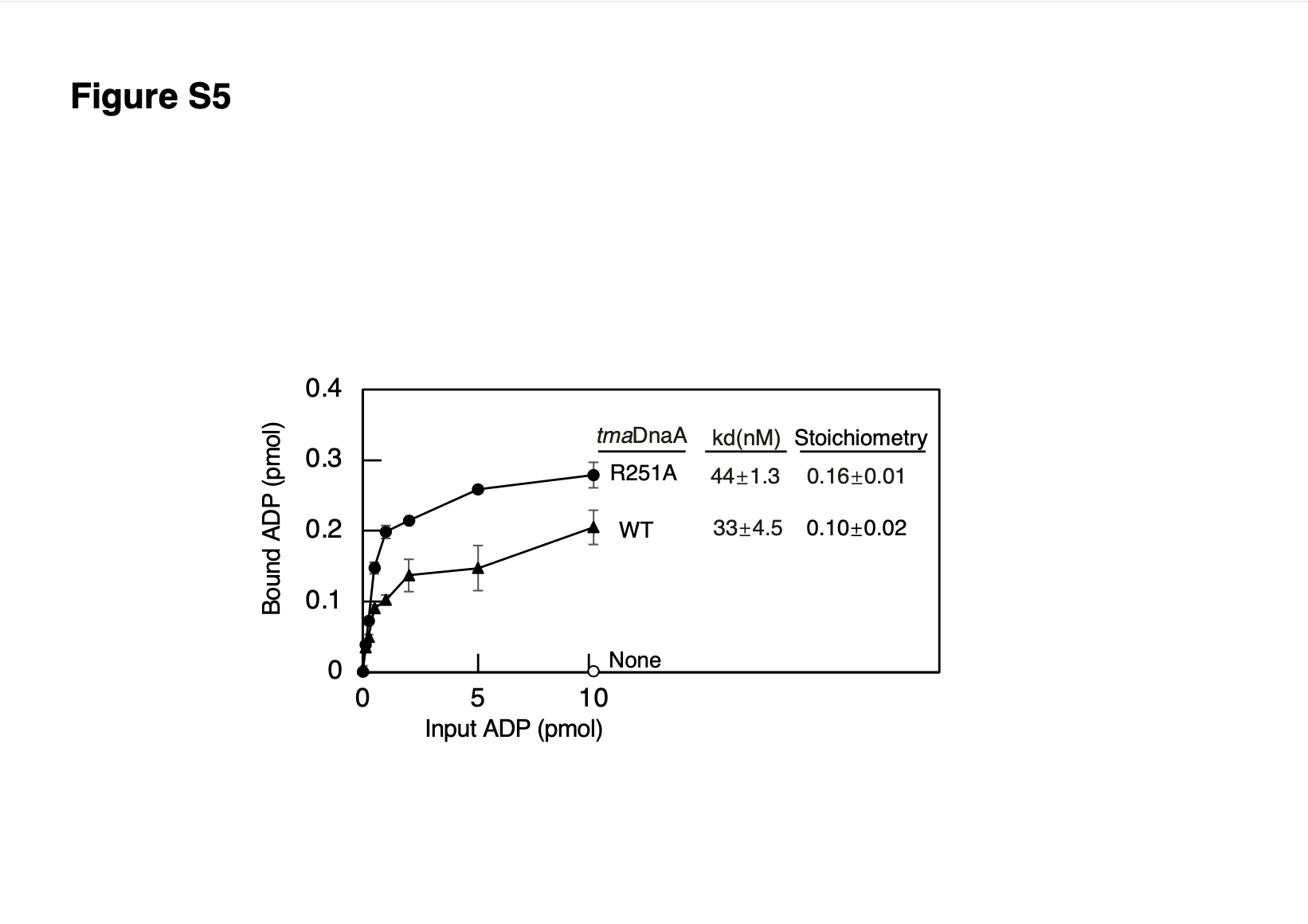


**Figure S5 ATP-binding activity of WT *tma*DnaA or *tma*DnaA R251A**

Wild-type (WT) *tma*DnaA or *tma*DnaA R251 was mixed with various concentrations of radio-labeled ADP, followed by filter retention assays. Mean ± standard deviation activity (n = 3). dissociation constant (Kd), and stoichiometry are shown as described in the legend to Figure 6.


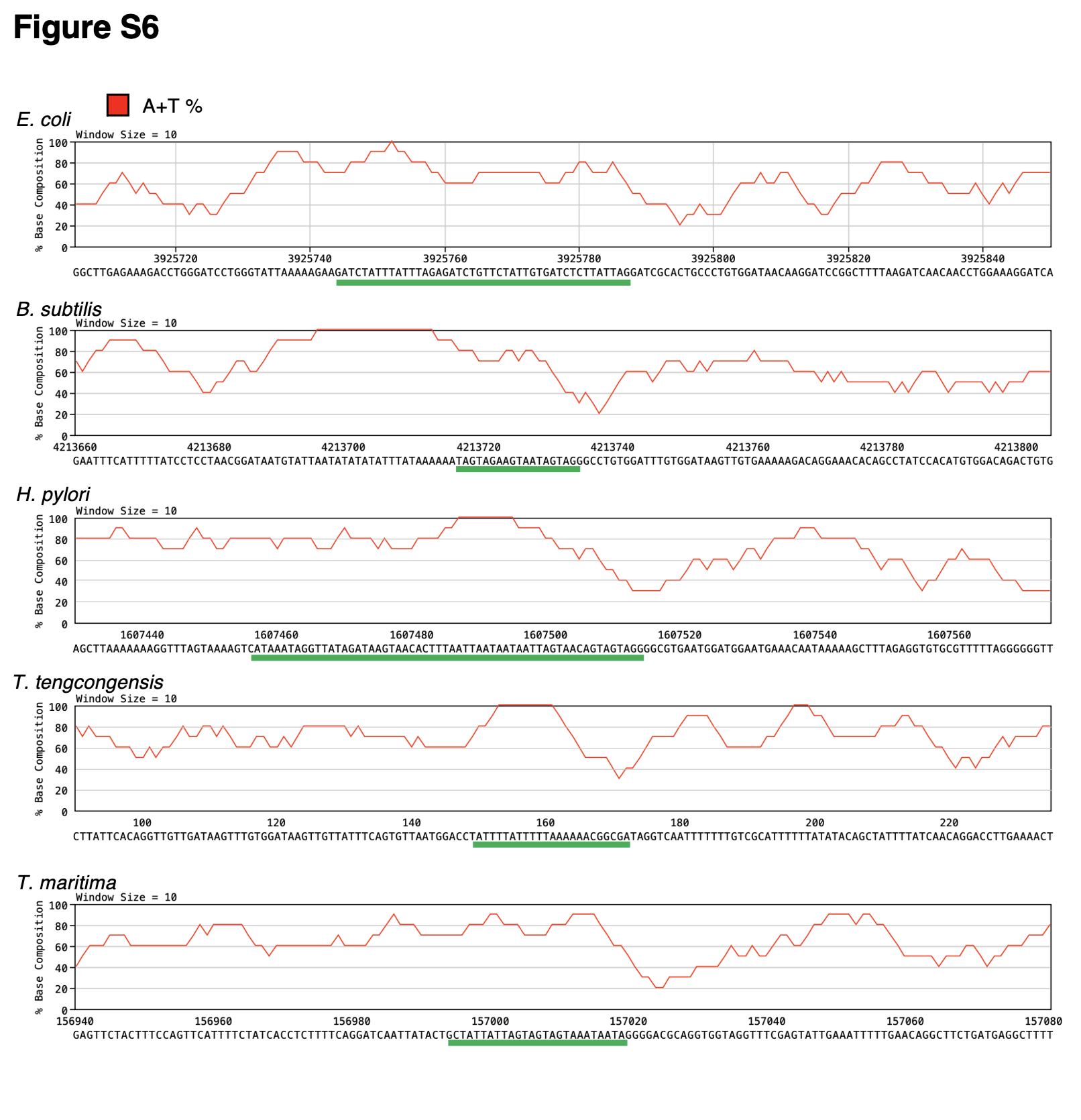


**Figure S6 The AT-content profiles**

The values of AT % for *E. coli*, *B. subtilis*, *H. pylori*, *T. tengcongensis*, and *T. maritima* genomes were plotted in a 10-bp sliding window using the MacVector software. DUE (green bars) and the flanking regions are shown. The x-axis indicates the genomic position of each organism.
